# Aberrant DNA Damage Response Pathways May Predict the Outcome of Platinum Chemotherapy in Ovarian Cancer

**DOI:** 10.1371/journal.pone.0117654

**Published:** 2015-02-06

**Authors:** Dimitra T. Stefanou, Aristotelis Bamias, Hara Episkopou, Soterios A. Kyrtopoulos, Maria Likka, Theodore Kalampokas, Stylianos Photiou, Nikos Gavalas, Petros P. Sfikakis, Meletios A. Dimopoulos, Vassilis L. Souliotis

**Affiliations:** 1 Institute of Biology, Medicinal Chemistry and Biotechnology, National Hellenic Research Foundation, 11635 Athens, Greece; 2 Department of Clinical Therapeutics, Athens University Medical School, 11528 Athens, Greece; 3 Genetic and Epigenetic Alterations of Genomes, de Duve Institute, Catholic University of Louvain, Brussels, 1200, Belgium; 4 Second Department of Obstetrics & Gynaecology, Athens University Medical School, 11528 Athens, Greece; 5 First Department of Propedeutic Medicine, Athens University Medical School, 11527 Athens, Greece; Garvan Institute of Medical Research, AUSTRALIA

## Abstract

Ovarian carcinoma (OC) is the most lethal gynecological malignancy. Despite the advances in the treatment of OC with combinatorial regimens, including surgery and platinum-based chemotherapy, patients generally exhibit poor prognosis due to high chemotherapy resistance. Herein, we tested the hypothesis that DNA damage response (DDR) pathways are involved in resistance of OC patients to platinum chemotherapy. Selected DDR signals were evaluated in two human ovarian carcinoma cell lines, one sensitive (A2780) and one resistant (A2780/C30) to platinum treatment as well as in peripheral blood mononuclear cells (PBMCs) from OC patients, sensitive (*n* = 7) or resistant (*n* = 4) to subsequent chemotherapy. PBMCs from healthy volunteers (*n* = 9) were studied in parallel. DNA damage was evaluated by immunofluorescence γH2AX staining and comet assay. Higher levels of intrinsic DNA damage were found in A2780 than in A2780/C30 cells. Moreover, the intrinsic DNA damage levels were significantly higher in OC patients relative to healthy volunteers, as well as in platinum-sensitive patients relative to platinum-resistant ones (all *P*<0.05). Following carboplatin treatment, A2780 cells showed lower DNA repair efficiency than A2780/C30 cells. Also, following carboplatin treatment of PBMCs *ex vivo*, the DNA repair efficiency was significantly higher in healthy volunteers than in platinum-resistant patients and lowest in platinum-sensitive ones (t_1/2_ for loss of γH2AX foci: 2.7±0.5h, 8.8±1.9h and 15.4±3.2h, respectively; using comet assay, t_1/2_ of platinum-induced damage repair: 4.8±1.4h, 12.9±1.9h and 21.4±2.6h, respectively; all *P*<0.03). Additionally, the carboplatin-induced apoptosis rate was higher in A2780 than in A2780/C30 cells. In PBMCs, apoptosis rates were inversely correlated with DNA repair efficiencies of these cells, being significantly higher in platinum-sensitive than in platinum-resistant patients and lowest in healthy volunteers (all *P*<0.05). We conclude that perturbations of DNA repair pathways as measured in PBMCs from OC patients correlate with the drug sensitivity of these cells and reflect the individualized response to platinum-based chemotherapy.

## Introduction

Ovarian cancer (OC) is the fifth most common type of cancer in females and the leading cause of mortality for gynecological malignancies, with epithelial carcinoma being the most frequent variety [1,2]. OC is typically diagnosed in advanced stages and often carries a dismal prognosis, even though current treatment strategies including aggressive surgical cytoreduction and platinum-paclitaxel chemotherapy seem to have significantly improved the relative survival of these patients to a 5-year survival rate of over 40% [3]. Established prognostic factors for OC include stage, histologic subtype, tumor grade and volume of residual disease after cytoreductive surgery [4,5].

Three different platinum compounds, namely cisplatin, carboplatin and oxaliplatin, are currently used in the clinical practice, with numerous indications, which cover a broad spectrum of solid tumors [6]. Their cytotoxic action is exerted through reaction with DNA and the development of DNA damage by the formation of cross-links, Pt-d[GpG] (1,2-intrastrand cross-links, 65%), Pt-d[ApG] (1,2-intrastrand cross-links, 25%) and in smaller extent Pt-d[GpNgG] (1,3-intrastrand cross-links), interstrand cross-links (ICLs, the most cytotoxic) and single-nucleotide damage of guanine [7]. Nucleotide excision repair (NER) is the main process by which platinum intrastrand crosslinks and single-nucleotide damage of guanine are repaired [8,9]. On the other hand, ICL repair is complex and requires a combination of NER, Fanconi Anemia repair pathway, translesion synthesis and homologous recombination [10–12]. Interestingly, ICLs repair proceeds via the formation of DNA double-strand breaks (DSBs) that represent the most lethal form of DNA damage [13]. The formation of DSBs is always followed by the phosphorylation of the histone H2AX, a variant of the H2A protein family, which is a component of the histone octamer in nucleosomes. The histone H2AX is phosphorylated by kinases such as ataxia telangiectasia mutated (ATM) and ATM-Rad3-related (ATR) in the PI3K pathway [14]. This newly phosphorylated protein, γH2AX, is the first step in recruiting and localizing DNA repair proteins.

The mammalian genome is protected against genotoxic insults by a network of DNA damage response (DDR) pathways, which are triggered by the detection of DNA lesions through specific sensors [15]. The subsequent step is the initiation of a signal transduction cascade, which activates various genome-protection pathways. Since DDR is a comprehensive signaling process that determines the cell fate either by repairing DNA damage or undergoing apoptosis, its role has been implicated in the disease process and in the success of chemotherapy. Indeed, it has been shown that abnormalities in the DNA repair pathways play an important role in the malignant transformation of OC [16]. Also, expression of DNA repair proteins, such as Breast Cancer 1 (BRCA1) and excision repair cross complementation group 1 (ERCC1) have correlated with poor survival in advanced OC and were found to be markers of resistance to platinum-based drugs [17–19]. In addition, our previous studies focused on the levels of DNA damage in peripheral blood mononuclear cells (PBMCs) from multiple myeloma patients have shown that DNA damage could prospectively distinguish between patients with different degrees of therapeutic response, providing the basis for pre-screening and selection of those patients more likely to benefit from this treatment [20–24].

Herein, we conducted a study to test the hypothesis that DDR, a crucial mechanism for cell survival, is involved in resistance to platinum chemotherapy. We found that OC patients are characterized by higher intrinsic DNA damage compared to healthy volunteers, and that the efficiency of DNA repair as measured in PBMCs from OC patients correlates with the drug sensitivity of these cells and reflects the individualized response to platinum-based chemotherapy.

## Materials and Methods

### Patients

All patients included in this analysis were managed in a single institution (Alexandra Hospital, Athens, Greece). Blood samples were obtained from eighteen (*n* = 18) patients undergoing surgery for suspected ovarian cancer (mean age 62 years, range 34–77) (Table 1) prior to any therapeutic treatment. Nine (*n* = 9) healthy individuals, age- and gender-matched to patients (all females, mean age 60 years, range 29–73) were served as controls. All patients were staged according to FIGO staging system. Chemotherapy was initiated within one month from surgery. Paclitaxel at 175mg/m^2^ over 3 hours immediately followed by carboplatin 5–6 AUC over 60 minutes were administered every 3 weeks. Six cycles of chemotherapy were administered. Seven patients did not receive post surgery chemotherapy: 3 had borderline tumors, 1 patient died in the postoperative period, while 3 patients refused any treatment after surgery. Platinum sensitivity for the 11 patients who received first line chemotherapy was assessed according to the Gynecologic Cancer Intergroup Committee (GCIC) criteria [25]. By these criteria, there were 4 platinum-resistant and 7 platinum-sensitive patients. Surviving patients should have a minimum follow-up of 2 years. All participants gave written informed consent according to the Declaration of Helsinki Principles, and the study was approved by the Institutional Review Board of Alexandra Hospital.

**Table 1:** Baseline characteristics of 18 patients with epithelial ovarian cancer included in this analysis.

Characteristic		No	% of total
Age (years)	Median (range)	62 (34–77)	
CA125 (U/ml)	Median (range)	304 (105–1295)	
PS	0	7	46.7
	1	5	33.3
	2	3	20
Histology	Serous	9	50
	Clear cell	6	33.3
	Borderline tumors	3	16.7
Grade	II	3	20
	III	12	80
Chemotherapy	Carboplatin/paclitaxel	11	61.1
Stage	I	1	6.7
	III	8	53.3
	IV	6	40
Surgical procedure	Ascites cytology only	1	6.7
	TAH^1^+BSO/USO^2^+Omentectomy	10	66.6
	Biopsy only	3	20
	Pelvic exenteration	1	6.7
Residual disease	0	2	13.3
	<2 cm	2	13.3
	2–5 cm	3	20
	>5 cm	5	33.4
	Unknown	3	20

^1^TAH: Total Abdominal Hysterectomy

^2^BSO/USO: Bilateral (or Unilateral) Salpingo-Oophorectomy

### Cell treatment

The platinum-sensitive A2780 and the platinum-resistant A2780/C30 cell lines [26] were kindly provided by Dr. George Koukos (Ovarian Cancer Research Center, University of Pennsylvania School of Medicine, Philadelphia, Pennsylvania, USA). Cells were cultured in monolayer by using RPMI 1640 containing 10% fetal calf serum, 100μg/ml streptomycin, 100units/ml penicillin, 0.3mg/ml glutamine and 0.3unit/ml insulin in a 37°C incubator continuously gassed with 5% CO_2_. Cells were treated with cisplatin (0–100μg/ml for 0–24h), followed by incubation in drug-free medium for 0–24h or carboplatin (0–300μg/ml for 0–24h), followed by post-incubation in drug-free medium for 0–24h.

PBMCs were isolated from freshly drawn peripheral blood using standard methods [23]. Then, cells were stimulated into proliferation using 10μg/ml phytohemagglutinin (PHA) for 48h at 37°C in RPMI 1640 supplemented with 10% FCS, 50mg/l penicillin, 50.000lU/l streptomycin and subsequently treated with cisplatin (0–300μg/ml for up to 3h), followed by incubation in drug-free medium for 0–24h or carboplatin (0–1800μg/ml for up to 24h), followed by post-incubation in drug-free medium for 0–24h.

### Cytotoxicity assay

Following drug treatment, viable cells were counted by trypan blue dye-exclusion [27]. Briefly, following incubation with the drug, cells were washed and resuspended in complete medium. An equal volume of 0.4% trypan blue reagent was added to the cell suspension and the percentage of viable cells was evaluated. Assays were performed in triplicate.

### Single-cell gel electrophoresis (Comet assay)

The single-cell gel electrophoresis assay was performed under alkaline conditions as described previously [28]. Briefly, aliquots of 5x10^4^ untreated or platinum drug treated cells were suspended in low melting point agarose (1%) in PBS (135mmol/l NaCl, 2.5mmol/l KCl, pH 10) at 37°C, and spread onto fully frosted microscope slides precoated with a thin layer of 1% normal melting agarose (Biozyme, Hameln, Germany). The cell suspension was immediately covered with a coverglass and the slides were kept at 4°C for 1h to allow solidification of the agarose. After removing the coverglass, cells were exposed to lysis buffer (2.5M NaCl, 100mM EDTA, 10mM Tris-HCI, pH 10, 1% Triton X-100) at 4°C for 1h. Then, the slides were placed in a horizontal gel electrophoresis chamber. The chamber was filled with cold electrophoresis buffer (1mM EDTA, 300mM NaOH, pH 13) and slides were kept at 4°C for 40min to allow the DNA to unwind. Electrophoresis was performed for 40min (1V/cm, 255mA). After electrophoresis, the slides were washed with neutralisation buffer (0.4M Tris-HCI, pH 7.5) for 10min and then with H_2_O for 10min. All preparative steps were conducted in dark to prevent additional DNA damage. The slides were stained with 20μl of 1μg/ml DAPI and analysed with a fluorescence microscope (NIKON Eclipse 400) equipped with a CCD-4230A video camera. Digital images were acquired using an image analysis system (Kinetic Analysis, Wirral, UK). Olive Tail Moments [OTM = (Tail Mean—Head Mean) x (% of DNA) / 100] of 100 cells/treatment condition were evaluated.

### Immunofluorescence antigen staining and confocal laser scanning microscope analysis

Aliquots of 2x10^4^ untreated or platinum drug treated cells were adhered to coverslip coated with 1M HCI and 50mg/ml poly-D-lysine prior to use, fixed by adding a 4% paraformaldehyde solution for 6min at room temperature and stored at -70°C until the analysis of pATM (serine-1981, Santa Cruz), pATR (serine-428, Cell Signaling), pChk1 (serine-345, Santa Cruz), pChk2 (threonine-68, Abcam) and γH2AX (serine-139, Cell Signaling) [29]. Cells were washed with cold PBS and blocked with 0.5ml per well blocking buffer (0.1% Triton X-100, 0.2% skimmed dry milk in PBS) for 1h at room temperature in a humidified box. Blocked cells were incubated with antibodies against pATM, pATR, pChk1, pChk2 or γH2AX in blocking buffer at 4°C overnight. After washing with blocking buffer, cells were incubated with goat anti-mouse antibody, FITC labeled, paired with goat anti-rabbit, TRITC labeled, for double labeling (Invitrogen) at a dilution of 1:4000 in blocking buffer for 1h at room temperature in the dark. Coverslips were applied, and the edges were sealed with clear nail polish. Images were visualized with a Leica TCS SP-1 confocal laser scanning microscope. Foci were manually counted in 200 cells/treatment condition and results are expressed as the % of γH2AX positive cells (mean±SD) from three independent experiments; positive cells are defined as cells with more than 5 foci per cell.

### Apoptosis assay

Apoptosis was evaluated by using the Cell Death Detection ELISA-PLUS kit (Roche Applied Sciences), according to manufacturer’s instructions. In brief, cells were treated with various doses of carboplatin (cell lines, 0–300μg/ml; PBMCs, 0–1800μg/ml) for 24h followed by incubation in drug-free medium for 24h. Then, cells were collected to prepare the cytosolic fractions that contained fragments of DNA. Equal volumes of these cytosolic fractions were incubated in anti-histone antibody-coated wells (96-well plates), and the histones of the DNA fragments were allowed to bind to the anti-histone antibodies. The peroxidase-labeled mouse monoclonal DNA antibodies were used to localize and detect the bound fragmented DNA using photometric detection with 2,29-azino-di-(3-ethylbenzthiazoline sulfonate) as the substrate. The test quantifies apoptosis as the fold increase (expressed as Enrichment Factor, EF) in the level of apoptosis in treated samples to untreated samples. We calculated the specific enrichment of mono- and oligo-nucleosomes released into the cytoplasm using the following formula: (EF) = [(absorbance of the treated cells) / (absorbance of the cells without drug treatment)]. Finally, the individual apoptosis rate was expressed as the carboplatin dose sufficient to trigger the induction of a certain Enrichment Factor (EF = 3).

### Statistical analysis

The efficiency of DNA repair and the induction of apoptosis were compared between groups of individuals using non-parametric tests. Specifically, Kruskal Wallis analysis was used for comparisons across all three groups, and Wilcoxon rank sum test for the pair wise comparisons. Correlations between the values of the various DDR-related parameters within the cell lines or PBMCs of the different OC patients were assessed by the Spearman correlation coefficient (all groups combined). To assess the linear association between DNA damage and platinum drug dose, apoptosis and DNA damage, as well as apoptosis and pan-nuclear staining, linear regression of these parameters was performed. A *P*-value less than 0.05 was considered statistically significant. To account for multiple comparisons the Bonferonni correction was used.

## Results

### DNA repair of platinum-induced damage in ovarian carcinoma cell lines correlates with the drug sensitivity of these cells

**Fig 1:**
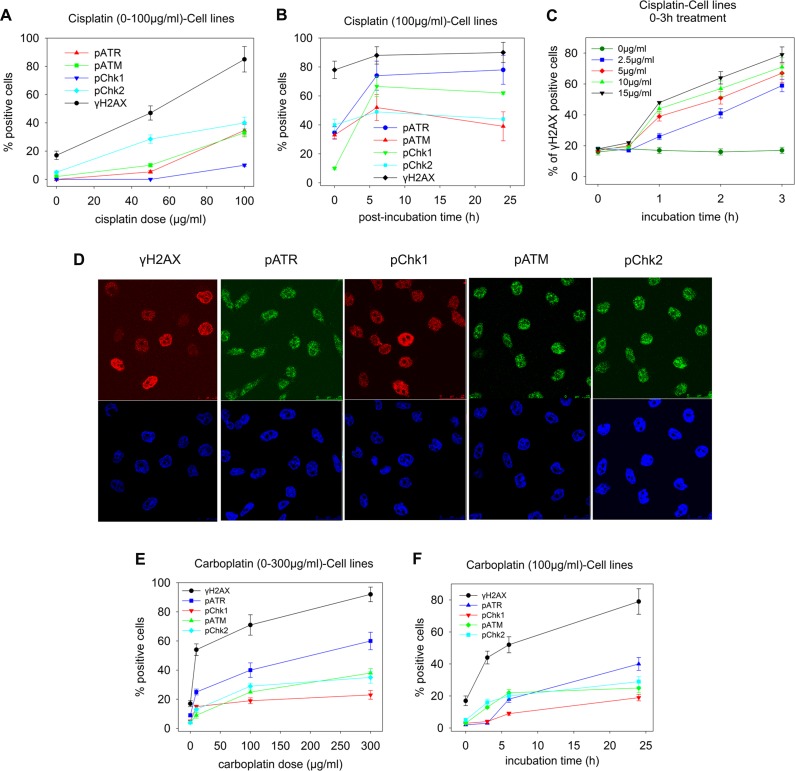
Changes in key molecules of the DDR pathways in ovarian carcinoma cell lines. A2780/C30 cells (representative of the two OC cell lines) were treated (A) with cisplatin (0–100μg/ml) for 3h, or (B) with 100μg/ml cisplatin, subsequently incubated in drug-free medium for various time-periods (0–24h), or (C) with relatively small doses (0–15μg/ml) of cisplatin for up to 3h, and analyzed at the end of the treatment using confocal microscopy. Positive cells: cells with more than 5 foci per cell. The error bars represent standard deviation. In (D) typical images showing the key molecules under study using microscope analysis of A2780/C30 cells treated with 100μg/ml of cisplatin for 3h; upper images, immunofluorescence antigen staining; bottom images, cell nuclei labeled with DAPI. (E) A2780/C30 cells were treated with carboplatin (0–300μg/ml) for 24h or (F) with 100μg/ml carboplatin for various time-periods (0–24h) and analyzed using confocal microscopy. The error bars represent standard deviation. All assays were performed in triplicate.

To examine the molecular mechanisms implicated in the drug sensitivity of platinum chemotherapy, changes in key molecules of the cellular DDR pathway (pATM, pATR, pChk1, pChk2, γH2AX) were evaluated in two ovarian carcinoma cell lines: one platinum-sensitive (A2780) and one platinum-resistant (A2780/C30) [26]. Cells were treated with various doses of cisplatin (0–100μg/ml) for 0–24h followed by incubation in drug-free medium for 0–24h. Both cell lines showed a dose-dependent increase in the formation of all key molecules under study (Fig. 1A, D). Maximal levels of these molecules were observed at 6h post-treatment, remaining stable or slightly decreasing thereafter (Fig. 1B). Interestingly, γH2AX showed the highest induction rates among the key molecules examined. The lowest cisplatin concentration at which γH2AX induction could be detected was 2.5μg/ml for 1h (Fig. 1C). Moreover, cells were treated with carboplatin (0–300μg/ml) for 0–24h followed by post-incubation in drug-free medium for 0–24h. Similar results to those obtained from the cisplatin experiments were found. That is, in both cell lines a dose-dependent increase in the formation of all key molecules was observed (Fig. 1E, F). Also, maximal levels of these molecules were observed at the end of the 24h treatment, decreasing thereafter. Again, the γH2AX showed the highest induction rates among the key molecules examined. Taken together, these results indicate that immunofluorescence quantification of γH2AX foci is a powerful approach to measure DNA damage induced by platinum drugs.

**Fig 2:**
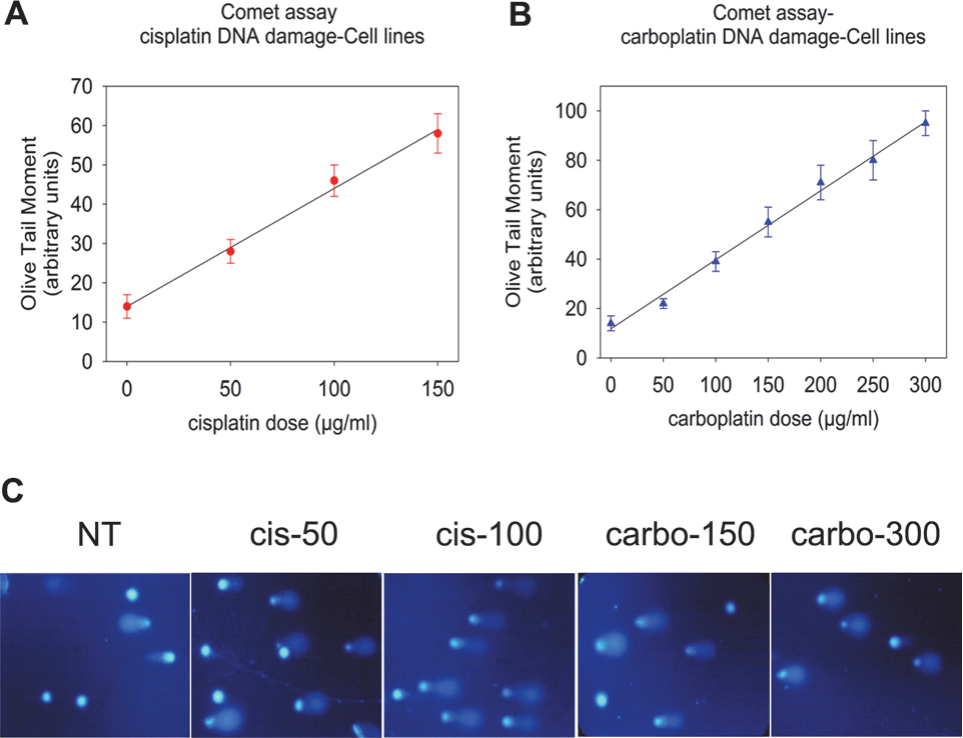
Measurement of DNA damage in ovarian carcinoma cell lines using alkaline comet assay. A2780/C30 cells (representative of the two OC cell lines) were treated with (A) cisplatin (0–150μg/ml) for 3h or (B) carboplatin (0–300μg/ml) for 24h, and analyzed at the end of the treatment using comet assay. The error bars represent standard deviation. In (C) typical comet assay images of A2780/C30 cells non-treated (NT), treated with 50μg/ml cisplatin (cis-50), 100μg/ml cisplatin (cis-100), 150μg/ml carboplatin (carbo-150) or 300μg/ml carboplatin (carbo-300). All assays were performed in triplicate.

The levels of platinum-induced DNA damage in both cell lines were also evaluated using alkaline comet assay. This version of the comet assay detects DNA migration caused by strand breaks, alkaline labile sites, and transient repair sites [30]. Following treatment of cells with 0–150μg/ml cisplatin for 3h (Fig. 2A, C) or 0–300μg/ml carboplatin for 24h (Fig. 2B, C), a linear dose-dependent increase of DNA damage levels was obtained in both cell lines, indicating that the method has the sensitivity and accuracy to detect and quantify platinum-induced DNA damage.

Furthermore, using the two powerful approaches validated above, the levels of the intrinsic DNA damage were assessed in untreated cell lines. Both immunofluorescence γH2AX staining and alkaline comet assay showed significant differences between the two cell lines, with the intrinsic DNA damage being higher in A2780 than in A2780/C30 cells. Particularly, using γH2AX immunofluorescence staining, A2780 cells exhibited 28.8±3.4% positive cells (i.e. cells with more than 5 foci per cell) and A2780/C30 17.4±3.4% positive cells (*P* = 0.01; Table 2). Moreover, by using comet assay, A2780 cells showed OTM values of 33.9±5.5 arbitrary units, while A2780/C30 cells showed 14.4±2.9 units (*P*<0.001; Table 2).

**Table 2:** DNA damage response-related parameters and associated Wilcoxon rank-sum tests in platinum-sensitive (A2780) and platinum-resistant (A2780/C30) cell lines.

Parameter measured	Method	Mean(SD)		*P*-values^9^
		A2780	A2780/C30	A2780 vs A2780/C30
Intrinsic DNA damage	γH2Ax	28.8(4.7)^1^	17.4(3.4)	0.01
	Comet assay	33.9(5.5)^2^	14.4(2.9)	<0.001
Induced DNA damage	γH2Ax	17200(3650)^3^	12400(2140)	0.01
	Comet assay	14900(3280)^4^	9400(1150)	0.01
DNA repair efficiency	γH2Ax	11.7(2.6)^5^	5.2(0.7)	<0.01
	Comet assay	16.7(3.4)^6^	9.9(1.3)	<0.01
Apoptosis	ELISA	43.8(5.6)^7^	78.5(9.2)	0.001
Pan-nuclear staining	γH2Ax	13400(2850)^8^	7500(1760)	0.001

^1^cells that are defined as having more than 5 foci per cell, expressed as a fraction of 100

^2^Olive Tail Moment (OTM) values, arbitrary units

^3^AUC values, expressed as (% of γH2AX positive staining cells) x (carboplatin dose)

^4^AUC values (expressed as OTM x carboplatin dose)

^5^t_1/2_ for γH2AX foci removal, in h

^6^t_1/2_ values for carboplatin-induced damage repair, in h

^7^apoptosis rates expressed as doses of carboplatin inducing apoptosis (as defined in the Materials and Methods section)

^8^AUC values, expressed as (% of pan-nuclear staining cells) x (carboplatin dose)

^9^Wilcoxon rank-sum tests.

To exhibit the DNA repair efficiency in these two cell lines, cells were treated with carboplatin (0–300μg/ml) for 24h followed by post-incubation in drug-free medium for 0–24h and the induced DNA damage (i.e. total DNA damage normalized by the intrinsic DNA damage) was analyzed at the end of drug treatment. Both immunofluorescence γH2AX staining and comet assay showed significant differences between the two cell lines. That is, in both cell lines DNA damage reached highest levels at the end of the drug treatment, with DNA damage being significantly higher in A2780 than in A2780/C30 cells (data not shown). Thereafter, carboplatin-induced DNA damage levels were reduced and the extent of repair was significantly lower in A2780 than in A2780/C30 cells (*P*<0.03). Particularly, using confocal microscopy the γH2AX foci were removed with t_1/2_ = 5.2±0.7h in A2780/C30 cells and 11.7±2.6h in A2780 cells. By using comet assay, the t_1/2_ values for carboplatin-induced damage repair in A2780/C30 and A2780 cells were 9.9±1.3h and 16.7±3.4h, respectively.

Moreover, the Area Under the Curve (AUC) for carboplatin-induced DNA damage, a parameter that reflects the overall DNA damage burden resulting from initial damage formation and DNA repair was calculated (Table 2). By using γH2AX immunofluorescence staining, A2780 cells showed AUC values [expressed as (% of γH2AX positive staining cells) x (carboplatin dose)] of 17200±3650, and A2780/C30 cells 12400±2140 (*P* = 0.01). Furthermore, comet assay confirmed the above findings with A2780 cells showing AUC values [expressed as (OTM x carboplatin dose)] of 14900±3280 while A2780/C30 cells showed AUC values of 9400±1150 (*P* = 0.01).

In addition, both cell types were treated with various doses of carboplatin (0–300μg/ml) for 24h and the induction of apoptosis was measured 24h post-treatment. We found that apoptosis rates were higher in the A2780 cells than in A2780/C30 cells (*P* = 0.001; Table 2). In particular, A2780 cells showed evidence of apoptosis at carboplatin doses as low as 43.8±5.6μg/ml, while A2780/C30 cells required a dose of 78.5±9.2μg/ml, indicating that the drug sensitivity of cells inversely correlated with their DNA repair capacity (Table 2).

A noteworthy finding was that following carboplatin treatment, a fraction of cells showed diffuse, bright pan-nuclear γH2AX staining. Previous studies have shown that pan-nuclear γH2AX staining represents a pre-apoptotic signal associated with ATM- and JNK-dependent apoptosis during replication [31]. In line with the results from the DNA repair efficiency experiments, we observed higher levels of pan-nuclear γH2AX staining [expressed as AUC (% of pan-nuclear staining cells) x (carboplatin dose)] in A2780 cells (13400±2850) than in A2780/C30 cells (7500±1760) (*P* = 0.001, Table 2).

### Deficient DNA repair of platinum-induced damage in PBMCs of OC patients correlates with better clinical outcome

**Fig 3:**
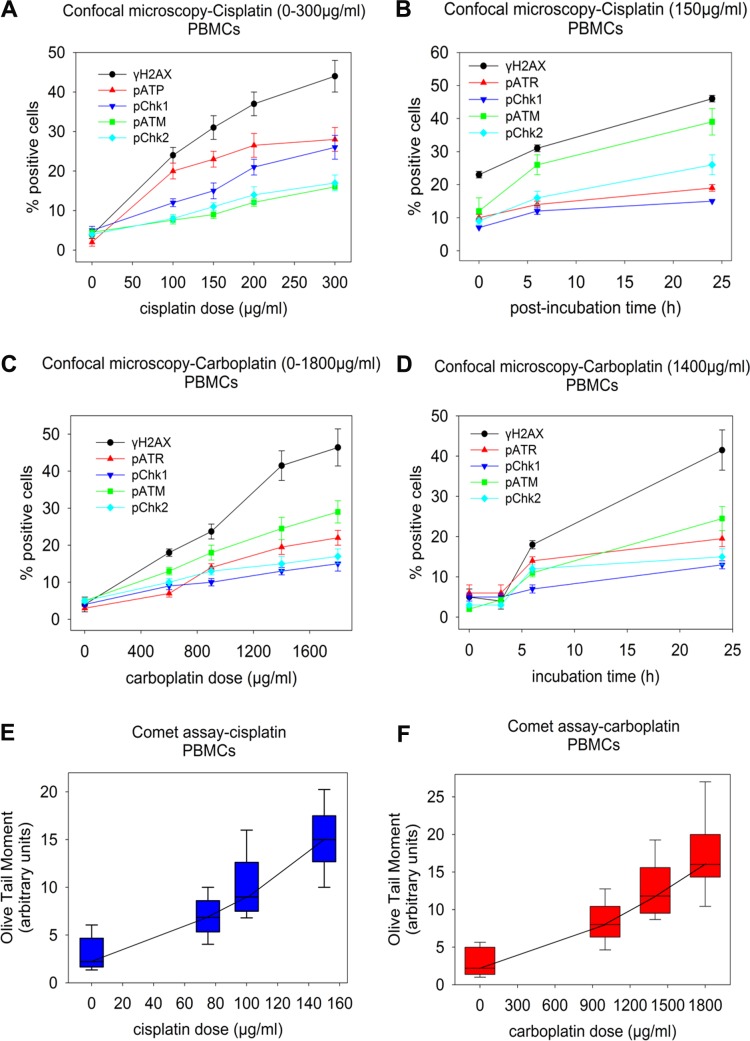
Changes in key molecules of the DDR pathways and comet assay in PBMCs from healthy volunteers. PBMCs from nine healthy volunteers were *ex vivo* treated (A) with cisplatin (0–300μg/ml) for 3h or (B) with 150μg/ml cisplatin, subsequently incubated in drug-free medium for various time-periods (0–24h), and analyzed thereafter using confocal microscopy. Also, PBMCs from the same healthy volunteers were treated (C) with carboplatin (0–1800μg/ml) for 24h or (D) with 1400μg/ml carboplatin for various times (0–24h) and analyzed at the end of the treatment using confocal microscopy. The error bars represent standard deviation. Finally, PBMCs were treated with (E) cisplatin (0–150μg/ml) for 3h or (F) carboplatin (0–1800μg/ml) for 24h and analyzed thereafter using comet assay. Box plots show statistical distribution of the levels of DNA damage. The horizontal lines within the boxes represent the median values and the vertical lines extending above and below the box indicate maximum and minimum values, respectively. All assays were performed in triplicate.

Turning to PBMCs, in agreement with previous studies [32], we found that following treatment of non-dividing PBMCs with platinum drugs, very low or null induction rates of the key molecules under study were observed. Therefore, we first treated PBMCs from nine healthy volunteers with various doses of PHA for up to 72h to induce proliferation of the cells. Optimal results were obtained following 48h incubation of PBMCs with 10μg/ml PHA (data not shown). Then, PBMCs were treated with cisplatin (0–300μg/ml for up to 3h) followed by incubation in drug-free medium for 0–24h or with carboplatin (0–1800μg/ml for up to 24h) followed by post-incubation in drug-free medium for 0–24h. In accordance to the results from the cell lines experiments, by either of the platinum drugs, a dose-dependent induction of all key molecules was observed (Fig. 3A, C), with the γH2AX again showing the highest induction rates (Fig. 3B, D). Moreover, the levels of platinum-induced DNA damage in PBMCs from the same nine healthy volunteers were measured using alkaline comet assay. We also found that the *ex vivo* treatment of PBMCs with cisplatin (Fig. 3E) or carboplatin (Fig. 3F) showed a dose-dependent increase of DNA damage levels. Taken together, the results from both cell lines and PBMCs experiments indicate that immunofluorescence quantification of γH2AX foci and alkaline comet assay are two powerful approaches for the quantification of platinum-induced DNA damage in clinical samples.

**Fig 4:**
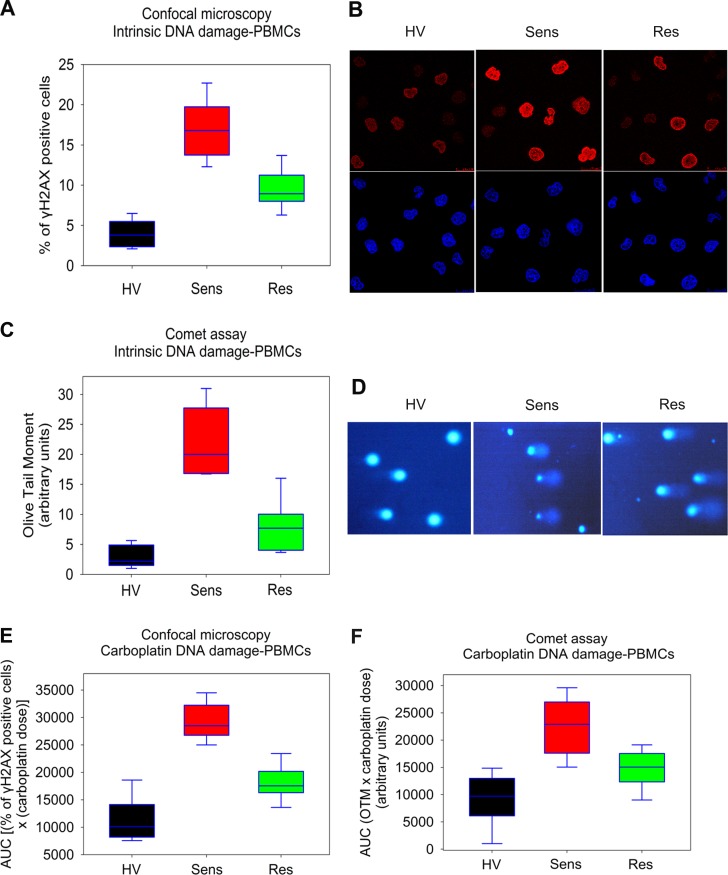
Intrinsic and carboplatin-induced DNA damage in PBMCs from healthy volunteers and OC patients. (A) Box plots showing statistical distribution of the levels of the intrinsic DNA damage in untreated PBMCs using immunofluorescence quantification of γH2AX. HV, healthy volunteers; Sens: OC patients sensitive to subsequent platinum therapy; Res: OC patients resistant to subsequent platinum therapy. (B) Typical images from confocal laser scanning microscope analysis of PBMCs from the three groups; upper images, γH2AX staining; bottom images, cell nuclei labeled with DAPI. (C) Box plots showing statistical distribution of the levels of the intrinsic DNA damage in untreated PBMCs using alkaline comet assay. (D) Typical comet images from untreated PBMCs of the three groups of individuals. Box plots showing statistical distribution of the levels of DNA damage in PBMCs stimulated into proliferation using PHA and treated with carboplatin (0–1800μg/ml) for 24h, using (E) immunofluorescence quantification of γH2AX and (F) alkaline comet assay. The horizontal lines within the boxes represent the median values and the vertical lines extending above and below the box indicate maximum and minimum values, respectively. All assays were performed in triplicate.

Thereafter, using these assays we examined the intrinsic levels of DNA damage in untreated PBMCs from the three groups of individuals (healthy volunteers, patients sensitive and patients resistant to platinum chemotherapy). Both γH2AX immunofluorescence staining and comet assay showed significant differences among the three groups of individuals (all *P*<0.05; Table 3), with the intrinsic DNA damage being higher in OC patients than in healthy volunteers. Interestingly, in line with the results from the ovarian carcinoma cell lines experiments, higher levels of intrinsic DNA damage were observed in PBMCs from patients sensitive compared to those resistant to subsequent platinum chemotherapy (γH2AX, *P* = 0.05; comet assay, *P* = 0.003; Table 3; Fig. 4A-D). Particularly, using γH2AX immunofluorescence staining, platinum-sensitive patients exhibited 16.8±2.3% positive cells, platinum-resistant patients 8.9±2.4%, while healthy volunteers showed 3.9±1.2% positive cells (Table 3; Fig. 4A). By using comet assay, patients sensitive to platinum chemotherapy showed OTM values of 20.1±5.5 arbitrary units, platinum-resistant 7.8±2.5, while healthy volunteers showed 2.4±1.1 units (Table 3; Fig. 4C). Interestingly, to confirm that the differences in the DDR signals are really reflective of platinum sensitivity rather than tumor type, patients bearing the same histotype were divided into sensitive and resistant to platinum therapy and the intrinsic DNA damage levels were compared. Although each subgroup contains a small number of samples, the results showed that differences in the intrinsic DNA damage levels are reflective of platinum sensitivity. For example, in serous tumors only, using γH2AX staining, sensitive patients (*n* = 6) showed higher levels of intrinsic DNA damage (mean value, 16.6% positive cells; range, 13.1–23.2%) than resistant patient (*n* = 1; 7.5%). In addition, in clear cell ovarian cancer only, sensitive patient (*n* = 1) exhibited 18.5% positive cells, while resistant patients (*n* = 3) only 9.4% (range, 6.3–13.7%). Similar results were obtained using comet assay.

**Table 3:** DNA damage response-related parameters and associated Wilcoxon rank-sum tests in PBMCs from healthy volunteers and OC patients, sensitive or resistant to platinum therapy.

Parameter measured	Method	Mean (range)			*P*-values^12^		
		HV^1^ (n = 9)	PS^2^ (n = 7)	PR^3^ (n = 4)	HV vs PS	HV vs PR	PS vs PR
Intrinsic DNA damage	γH2Ax	3.9^4^	16.8	8.9	<0.01	0.04	0.05
		2.1–6.5	13.1–23.2	6.3–13.7			
	Comet assay	2.4^5^	20.1	7.8	<0.001	<0.01	0.003
		1.1–5.8	16.7–31.1	3.7–16.1			
Induced DNA damage	γH2Ax	10300^6^	28500	17700	0.02	0.01	0.03
		7500–18700	25100–34400	13700–23500			
	Comet assay	9850^7^	22700	16240	<0.01	<0.01	0.05
		1050–14930	14990–29640	6840–21870			
DNA repair efficiency	γH2Ax	2.7^8^	15.4	8.8	0.03	0.03	<0.01
		1.5–3.2	12.8–18.2	6.1–10.6			
	Comet assay	4.8^9^	21.4	12.9	0.02	0.01	<0.01
		2.3–6.7	17.3–26.0	8.5–15.2			
Apoptosis	ELISA	1597^10^	605	1020	<0.01	<0.05	<0.01
		1350–2000	390–1050	800–1370			
Pan-nuclear staining	γH2Ax	4600^11^	20700	11100	<0.001	<0.005	<0.01
		850–9500	15900–27000	7300–19400			

^1^HV, Healthy volunteers

^2^PS, Platinum-sensitive patients

^3^PR, Platinum-resistant patients

^4^PBMCs that are defined as having more than 5 foci per cell, expressed as a fraction of 100

^5^Olive Tail Moment (OTM) values, arbitrary units

^6^AUC values, expressed as (% of γH2AX positive staining cells) x (carboplatin dose)

^7^AUC values (expressed as OTM x carboplatin dose)

^8^t_1/2_ for γH2AX foci removal, in h

^9^t_1/2_ values for carboplatin-induced damage repair, in h

^10^apoptosis rates expressed as doses of carboplatin inducing apoptosis (as defined in the Materials and Methods section)

^11^AUC values, expressed as (% of pan-nuclear staining cells) x (carboplatin dose)

^12^Wilcoxon rank-sum tests

To examine the DNA repair efficiency in the three groups of individuals, following 48h incubation of PBMCs with 10μg/ml PHA, cells were treated with carboplatin (0–1800μg/ml) for 24h, post-incubated in drug-free medium for 0–24h and the induced DNA damage was analyzed. Both γH2AX immunofluorescence staining and comet assay showed similar results. That is, in all individuals analyzed DNA damage reached highest levels at the end of the drug treatment, with DNA damage being higher in OC patients than in healthy volunteers; platinum-sensitive patients showed higher levels of DNA damage than platinum-resistant ones (data not shown). Thereafter, DNA damage levels were eliminated and the extent of repair was higher in healthy volunteers than in patients. Interestingly, in line with the results from the cell lines experiments, platinum-sensitive patients showed significantly lower repair efficiency than platinum-resistant ones (*P*<0.03). Particularly, using confocal microscopy the γH2AX foci were removed with t_1/2_ = 2.7±0.5h in healthy controls, 8.8±1.9h in platinum-resistant and 15.4±3.2h in platinum-sensitive patients. By using comet assay, the t_1/2_ values for carboplatin-induced DNA damage repair were 4.8±1.4h, 12.9±1.9h and 21.4±2.6h, respectively. Notably, in serous tumors only, the γH2AX foci were removed with t_1/2_ = 15.4h in sensitive patients (*n* = 6; range, 12.8–18.2h) and t_1/2_ = 8.4h in the resistant one (*n* = 1). In addition, in clear cell ovarian cancer only, sensitive patient (*n* = 1) showed t_1/2_ = 17.1h, while resistant patients t_1/2_ = 8.9h (*n* = 3; range, 6.1–10.6h). Similar results were obtained using comet assay.

The AUC levels for carboplatin-induced DNA damage were also calculated. By using confocal microscopy, platinum-sensitive patients showed AUC values of 28500±2323, platinum-resistant patients 17700±1138, while healthy volunteers showed values of 10300±810 (Table 3, all *P*<0.05) (Fig. 4E). Comet assay showed similar results. That is, patients sensitive to chemotherapy showed AUC values of 22700±540, platinum-resistant patients 16240±2417, while healthy volunteers showed AUC values of 9850±540 (Table 3; Fig. 4F). Interestingly, in serous tumors only, sensitive patients showed AUC values of 28400 (*n* = 6; range, 25100–34400), while the platinum-resistant patient 15200 (*n* = 1). In addition, in clear cell ovarian cancer only, sensitive patient (*n* = 1) exhibited AUC values of 29000, while resistant patients (*n* = 3) only 18500 (range, 13700–23500). Similar results were obtained using comet assay.

**Fig 5:**
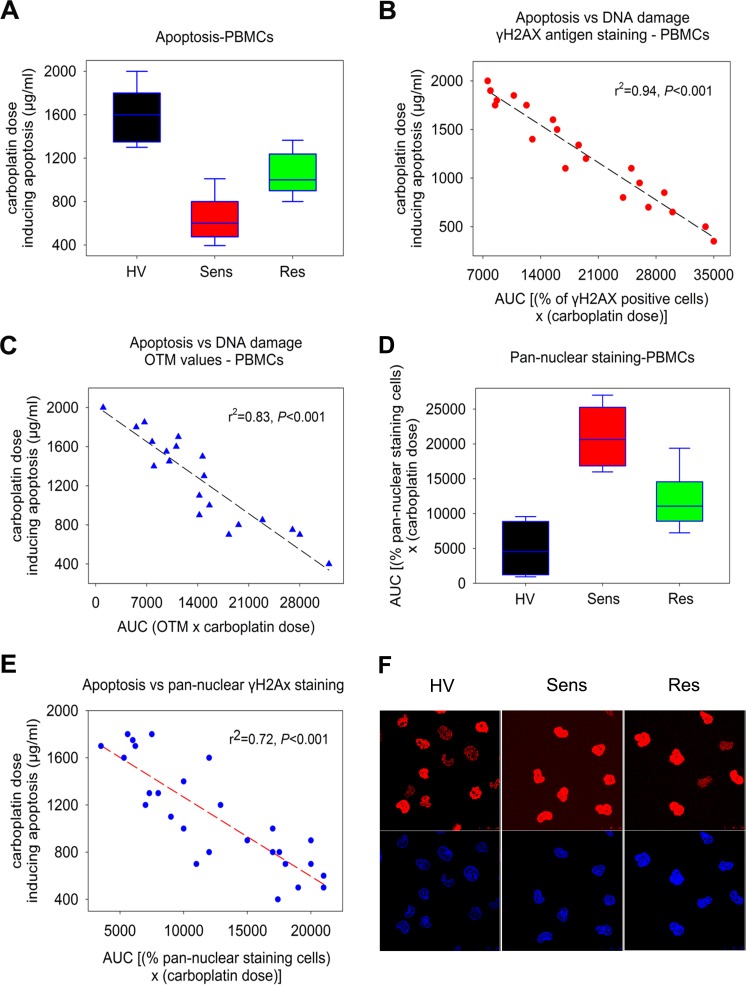
The induction of the apoptotic pathway in carboplatin-treated PBMCs. (A) Box plots showing statistical distribution of the individual apoptosis rates, expressed as doses of carboplatin inducing apoptosis in the three groups of individuals. HV, healthy volunteers; Sens: OC patients sensitive to subsequent platinum therapy; Res: OC patients resistant to subsequent platinum therapy. The correlations between the individual apoptosis rates and the DNA damage in PBMCs from the same individuals using γH2AX immunofluorescence staining (B) and comet assay (C) are presented. (D) Box plots showing statistical distribution of the levels of the pan-nuclear immunofluorescence γH2AX staining in the three groups of individuals. The horizontal lines within the boxes represent the median values and the vertical lines extending above and below the box indicate maximum and minimum values, respectively. (E) Correlation between pan-nuclear γH2AX staining and the individual apoptosis rates in the same samples. (F) Typical images showing pan-nuclear immunofluorescence γH2AX staining; upper images, γH2AX staining; bottom images, cell nuclei labeled with DAPI. All assays were performed in triplicate.

Moreover, following 48h incubation of PBMCs from the three groups of individuals with 10μg/ml PHA, cells were treated *ex vivo* with various doses of carboplatin (0–1800μg/ml) for 24h and the induction of apoptosis was measured 24h after the end of the treatment. In accordance with the results from the cell lines experiments, we found that apoptosis rates were higher in platinum-sensitive patients than in platinum-resistant ones, with healthy volunteers showing the lowest rates (all *P*<0.05; Fig. 5A). In particular, PBMCs from platinum-sensitive patients showed evidence of apoptosis at carboplatin doses of 605±36μg/ml, platinum-resistant patients at 1020±158μg/ml, while healthy volunteers at 1597±254μg/ml. Interestingly, an inverse correlation was observed between the apoptosis rates and the DNA repair efficiencies of the same individuals (linear regression analysis; microscope analysis: r^2^ = 0.94, *P*<0.001, Fig. 5B; comet assay: r^2^ = 0.83, *P*<0.001, Fig. 5C). Notably, in serous tumors only, sensitive patients (*n* = 6) showed evidence of apoptosis at carboplatin doses of 636μg/ml (range, 390–1050μg/ml) and the platinum-resistant patient at 1370μg/ml (*n* = 1). In addition, in clear cell ovarian cancer only, the corresponding values were 420μg/ml for the sensitive patient (*n* = 1) and 877μg/ml (*n* = 3; range, 800–940μg/ml) for the resistant ones.

Finally, using confocal microscope analysis, in agreement with the results from the apoptosis experiments we observed higher levels of pan-nuclear γH2AX staining in OC patients than in healthy volunteers (Fig. 5D, F). Particularly, patients sensitive to platinum chemotherapy showed higher levels of pan-nuclear staining expressed as AUC (20700±3143), than platinum-resistant patients (11100±1012; *P*<0.01; Table 3). Healthy volunteers showed the lowest levels of pan-nuclear staining (4600±477).

## Discussion

Mammalian cells have evolved a sophisticated signal transduction network to sense DNA damage and to mount an appropriate DDR pathway. Dysregulation of the DDR pathway has been involved into both malignant transformation and response to chemotherapy with DNA damaging drugs. Therefore, we conducted a study to shed light on the DDR signaling in OC patients, sensitive or resistant to platinum chemotherapy. We found that OC patients are characterized by higher intrinsic DNA damage compared to healthy volunteers, and that the efficiency of DNA repair as measured in PBMCs from OC patients inversely correlates with the response of individual patients to platinum-based chemotherapy.

Platinum-based drug cytotoxicity is mainly mediated by the introduction of ICLs into the DNA of treated cells. ICLs involve the covalent linking of the two strands of DNA which prevents their separation and consequently blocks transcription, segregation and replication. Repair of ICLs caused by platinum drugs involves the generation of DSBs as an intermediate step followed by the phosphorylation of the histone H2AX [33–35]. In the present study, by using confocal microscope analysis we first investigated changes in the expression of key molecules of the DDR pathways (pATM, pATR, pChk1, pChk2, γH2AX) after treatment of ovarian carcinoma cell lines and PBMCs from healthy volunteers with platinum-based drugs. In all cell types analyzed, a dose-dependent induction of all molecules under study was observed, with γH2AX showing the highest induction rates. Moreover, using ovarian carcinoma cell lines and PBMCs from healthy volunteers, we validated the comet assay, a technically simple and fast method that detects genotoxicity in virtually any mammalian cell type [36,37]. Thereafter, using these two powerful approaches the levels of the DNA damage were assessed in clinical samples.

Previous reports have shown a link between ovarian carcinogenesis and the repair efficiency of DNA damage [16]. In fact, defects in genes involved in DSBs repair, such as ERCC1, BRCA1 and BRCA2 are implicated in familial OC [17–19]. Also, a strong association between the MGMT (O^6^-methylguanine-DNA methyltransferase) activity and OC risk has been described [38]. In addition, the repair efficiency of the DNA damage induced by the alkylating agent MNNG (N-methyl-N-nitro-N-nitrosoguanidine) decreased with progression of the cervical cancer in a stepwise manner [39]. In the present study, we found that the intrinsic DNA damage was higher in OC patients than in healthy volunteers. Moreover, in line with the findings from the two OC cell lines, the intrinsic DNA damage was significantly higher in platinum-sensitive than in platinum-resistant patients. High intrinsic damage could be the outcome of increased formation of DNA damage and/or a delay in the repair of these lesions. Increased DNA damage formation can be generated by pathological or physiological agents or processes. These agents or processes can be exogenous such as ionizing radiation or endogenous such as oxidative free radicals, replication across a nick, inadvertent enzyme action at fragile sites, mechanical shearing at anaphase bridges, metabolic byproducts, and so forth [40–42]. Physiological processes such as V(D)J recombination, class switch recombination (CSR) and meiosis also induce DNA damage in the genome [43–45].

Since the higher intrinsic DNA damage gives no information about the repair capacity of the cells, we analyzed the repair efficiency of the carboplatin-induced DNA damage in both PBMCs and OC cell lines. In agreement with the results from the intrinsic DNA damage experiments, we found higher DNA repair efficiency in healthy volunteers than in OC patients, suggesting that malignant transformation correlates with a reduction of DNA repair efficiency. Whether reduced DNA repair is the cause or effect of carcinogenesis is still unclear. In any case, since failure to repair DNA damage can lead to genomic instability, the accumulation of DNA damage in OC patients may be involved in the pathogenesis of the disease.

Interestingly, the OC cell line sensitive to platinum treatment showed higher levels of DNA damage than the platinum-resistant one. In line with this finding, the levels of the *ex vivo* platinum-induced DNA damage in PBMCs were higher in platinum-sensitive than in platinum-resistant patients, reflecting the clinical response of OC patients to the subsequent chemotherapy (Table 3). Moreover, in agreement with the results from the DNA repair experiments, following treatment of the two OC cell lines with carboplatin, the apoptosis rates inversely correlated with the repair capacity of these cells, being higher in the sensitive than in the resistant cells. In line with this finding, following treatment of PBMCs with carboplatin, an inverse correlation was observed between the apoptosis rates and the DNA repair efficiencies of the same individuals, with the apoptosis rates being significantly higher in platinum-sensitive than in platinum-resistant patients and lowest in healthy volunteers (all *P*<0.01; Table 3). Moreover, in accordance to previous studies [31,46], the correlation between apoptosis and pan-nuclear γH2AX staining in PBMCs from the same patients confirm that pan-nuclear γH2AX staining represents a pre-apoptotic signal (linear regression analysis; r^2^ = 0.72, *P*<0.001, Fig. 5E).

It is generally accepted that ovarian cancer is not a single disease but is made up of several different distinct histotypes. The main histotypes are epithelial in origin and include high-grade serous carcinoma, clear cell carcinoma, endometrioid carcinoma, low-grade serous carcinoma, and mucinous carcinoma [47]. Also, there are several rare types of non-epithelial ovarian cancers including germ cell tumors, stromal tumors such as granulosa cell tumor and Sertoli Leydig cell tumor [48]. On the other hand, cell lines derived from tumors are the most frequently utilized models in *in vitro* drug sensitivity studies. In the present study, the cisplatin-sensitive A2780 cell line (the second most commonly used ovarian cancer cell line) [49], and the cisplatin-resistant cell line A2780/C30 (derived from A2780 following continuous exposure to increasing concentrations of cisplatin) [26] were examined. Owing to the molecular heterogeneity of the various histotypes of epithelial ovarian cancer, we recognize that a single pair of cell lines may not accurately reflect the broad spectrum of phenotypes and that use of patient-derived cells would enhance the study.

The results presented in the present study showed that the disease-associated nature of the defects in DDR pathways reflected in cells of the peripheral blood of OC patients. In agreement with these results, previous reports have shown that biomarkers (e.g. transcriptomic and epigenetic profiles, chromosomal aberrations, telomere length, DNA damage response signals) measured in peripheral blood leukocytes (a) can distinguish between healthy controls and subjects with a variety of diseases including various types of cancer [50–56] and (b) can predict clinical response to therapeutic treatment [20–23,57–59]. Although one cannot rule out the possibility that these findings may be caused by the presence in blood of circulating tumor cells [60,61], the very small numbers of such cells make such an explanation unlikely. Therefore, it seems possible that the regulation at the individual level of cellular responses to various influences is partly controlled by genetically or environmentally determined systemic factors and that, as regards response to the highly toxic effects of cytotoxic drugs, such systemic factors remain predominant despite any disease-related perturbations occurring in cancer cells.

Taken together, the results reported here demonstrate the critical importance of cellular responses to DNA damage in determining the cytotoxicity of platinum-based drugs, as measured in *in vitro* cellular systems, as well as their therapeutic efficiency in OC patients. In particular, we have found that the efficiency of DNA repair as measured in PBMCs from OC patients correlates with the drug sensitivity of these cells and reflects the response of individual patients to platinum-based chemotherapy. Given that these data were obtained in a readily accessible tissue (PBMCs) taken prior to any therapeutic treatment, if they are confirmed in a larger study, they may eventually be translated into novel indicators for the selection of patients who are more likely to benefit from platinum chemotherapy.
