# Correction: Aberrant DNA Damage Response Pathways May Predict the Outcome of Platinum Chemotherapy in Ovarian Cancer

**DOI:** 10.1371/journal.pone.0256051

**Published:** 2021-08-05

**Authors:** Dimitra T. Stefanou, Aristotelis Bamias, Hara Episkopou, Soterios A. Kyrtopoulos, Maria Likka, Theodore Kalampokas, Stylianos Photiou, Nikos Gavalas, Petros P. Sfikakis, Meletios A. Dimopoulos, Vassilis L. Souliotis

After this article [1] was published, the following data reporting issues came to light.

- In Fig 1D, the pATM data were duplicated in error in the pChk2 panel. Also, this figure did not include image data for the control (untreated) cells. These issues have been addressed in the updated Fig 1 provided with this notice.
- Several statements in the article describe A2780 cell results and/or compare A2780 versus A2780/C30 results, yet many of the A2780 cell results described were not reported in the article or public dataset. The A2780 data have been added to the Helios dataset (files “Additional data–Stefanou et al PLOS ONE 2014” Excel file pp. 1–8, 10, “A2780 cell line—confocal images—Stefanou et al PLOS ONE 2014”, and “A2780 cell line- comet images—Stefanou et al PLOS ONE 2014”), and representative results are included in S1 and S2 Figs.
- Fig 2C did not show image data for all doses represented in the graphs or indicate whether the NT image is from the cisplatin or from the carboplatin experiment. Image data for the full dose response series is provided in S3 Fig. The authors clarified that the NT image shown in the figure is from the cisplatin experiment, and that the non-treated cells yielded similar results in the cisplatin and carboplatin experiments.
- Fig 3B and 3D did not report control data showing expression levels throughout the timecourse in non-treated cells. Control data for these experiments have been provided in S4 File and in the “Additional data” Excel file (pp. 12–13) in the Helios dataset.
- The Results section includes a discussion of results for serous tumors versus clear ovarian cancers, but aside from the quantitative summary results in the text these subgroup data were not reported in the article. Results to support these statements are in S5 File.
- S6 File of this notice includes representative image data demonstrating the results shown in graph form in Fig 3. Supporting data for the results shown in Figs 4E, 4F and 5A-5C are also included. Quantitative data for experiments reported in Figs 3–5 are available in the Helios dataset.
- There are five statements in the article that refer to data not shown, which is not allowed per *PLOS ONE*’s Data Availability policy. The data underlying these statements are in S7 File and at the Helios repository.

A member of *PLOS ONE*’s Editorial Board reviewed the data files and the updated figure and advised that the concerns have been satisfactorily addressed.

**Fig 1:**
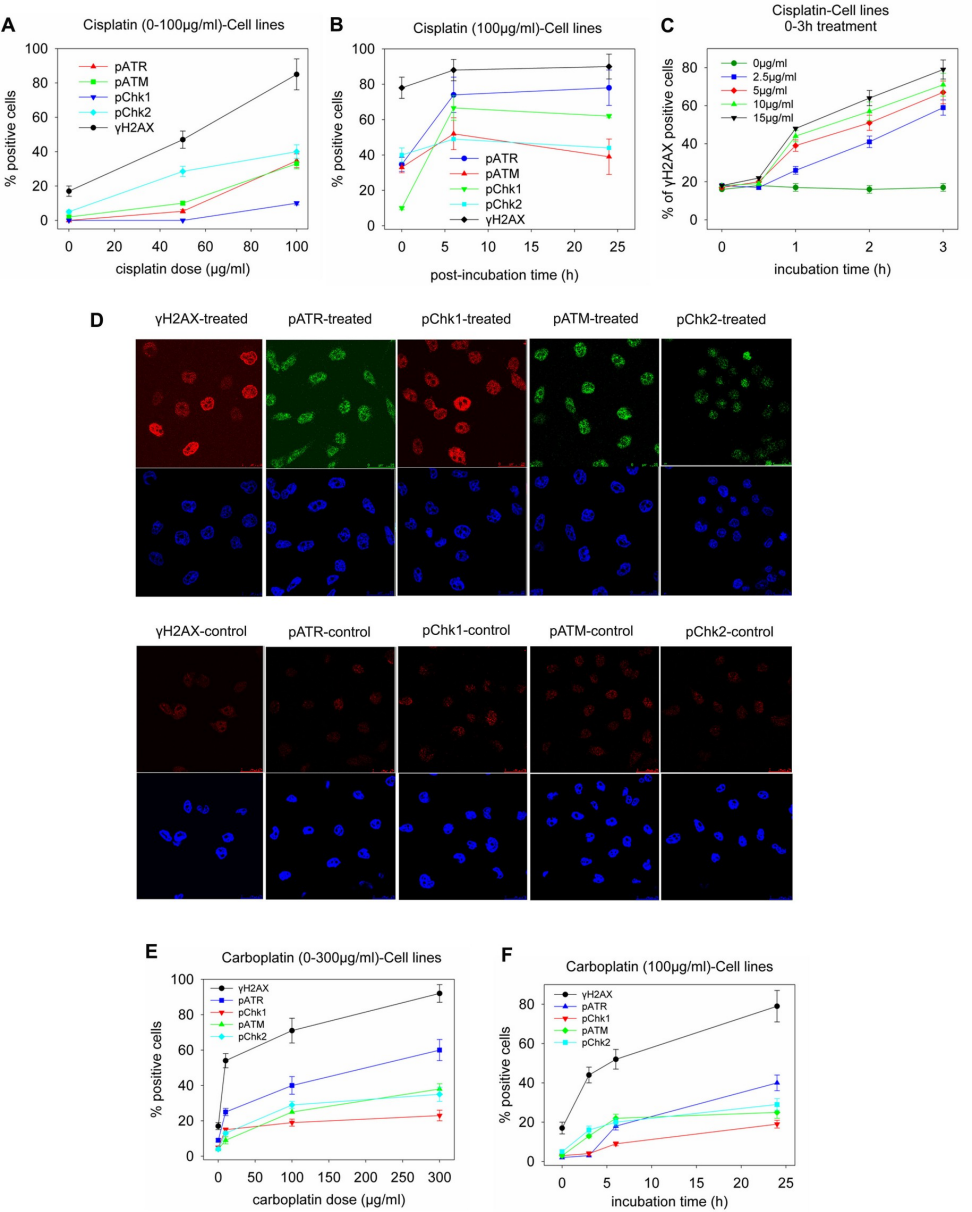
Changes in key molecules of the DDR pathways in ovarian carcinoma cell lines. A2780/C30 cells (representative of the two OC cell lines) were treated (A) with cisplatin (0–100μg/ml) for 3h, or (B) with 100μg/ml cisplatin, subsequently incubated in drug-free medium for various time-periods (0–24h), or (C) with relatively small doses (0–15μg/ml) of cisplatin for up to 3h, and analyzed at the end of the treatment using confocal microscopy. Positive cells: cells with more than 5 foci per cell. The error bars represent standard deviation. In (D) typical images showing the key molecules under study using microscope analysis of A2780/C30 cells treated with 100μg/ml of cisplatin for 3h; upper images, immunofluorescence antigen staining; bottom images, cell nuclei labeled with DAPI. Typical images showing the same key molecules in the control/untreated A2780/C30 cells are also presented. (E) A2780/C30 cells were treated with carboplatin (0–300μg/ml) for 24h or (F) with 100μg/ml carboplatin for various time-periods (0–24h) and analyzed using confocal microscopy. The error bars represent standard deviation. All assays were performed in triplicate.

## Supporting information

S1 Fig Changes in key molecules of the DDR pathways in the A2780 carcinoma cell line. (A2780 data corresponding to results shown in Fig 1D) Typical images showing the key molecules under study using microscope analysis of A2780 cells treated with 100 μg/ml cisplatin for 3h; upper images, immunofluorescence antigen staining; bottom images, cell nuclei labeled with DAPI. The key molecules of control/non-treated A2780 cells are also presented.
(JPG)

S2 Fig Measurement of DNA damage in the A2780 carcinoma cell line using alkaline comet assay. (A2780 data corresponding to results shown in Fig 2C) Typical comet assay images of A2780 cells treated with cisplatin (0–150 μg/ml) for 3h or carboplatin (0–300 μg/ml) for 24h and analyzed at the end of the treatment. NT, non-treated.
(JPG)

S3 Fig Measurement of DNA damage in the A2780/C30 carcinoma cell line using alkaline comet assay. (A2780/C30 data corresponding to results shown in Fig 2C) Typical comet assay images of A2780/C30 cells treated with cisplatin (0–150 μg/ml) for 3h or carboplatin (0–300 μg/ml) for 24h and analyzed at the end of the treatment. NT, non-treated.
(JPG)

S4 File Control data corresponding to results shown in Fig 3B and 3D. (DOC)

S5 File Results for serous tumors and clear ovarian cancers. (DOC)

S6 File Supporting data for the results shown in Figs 3, 4E, 4F and 5A-5C. (ZIP)

S7 File Results to support statements for which data were not shown in the original article. (ZIP)
