# Supplementary figures and images for: Correction: Aberrant DNA Damage Response Pathways May Predict the Outcome of Platinum Chemotherapy in Ovarian Cancer
Source: PLoS One. 2021 Aug 5;16(8):e0256051. doi: 10.1371/journal.pone.0256051 (PMC8341518; doi:10.1371/journal.pone.0256051)

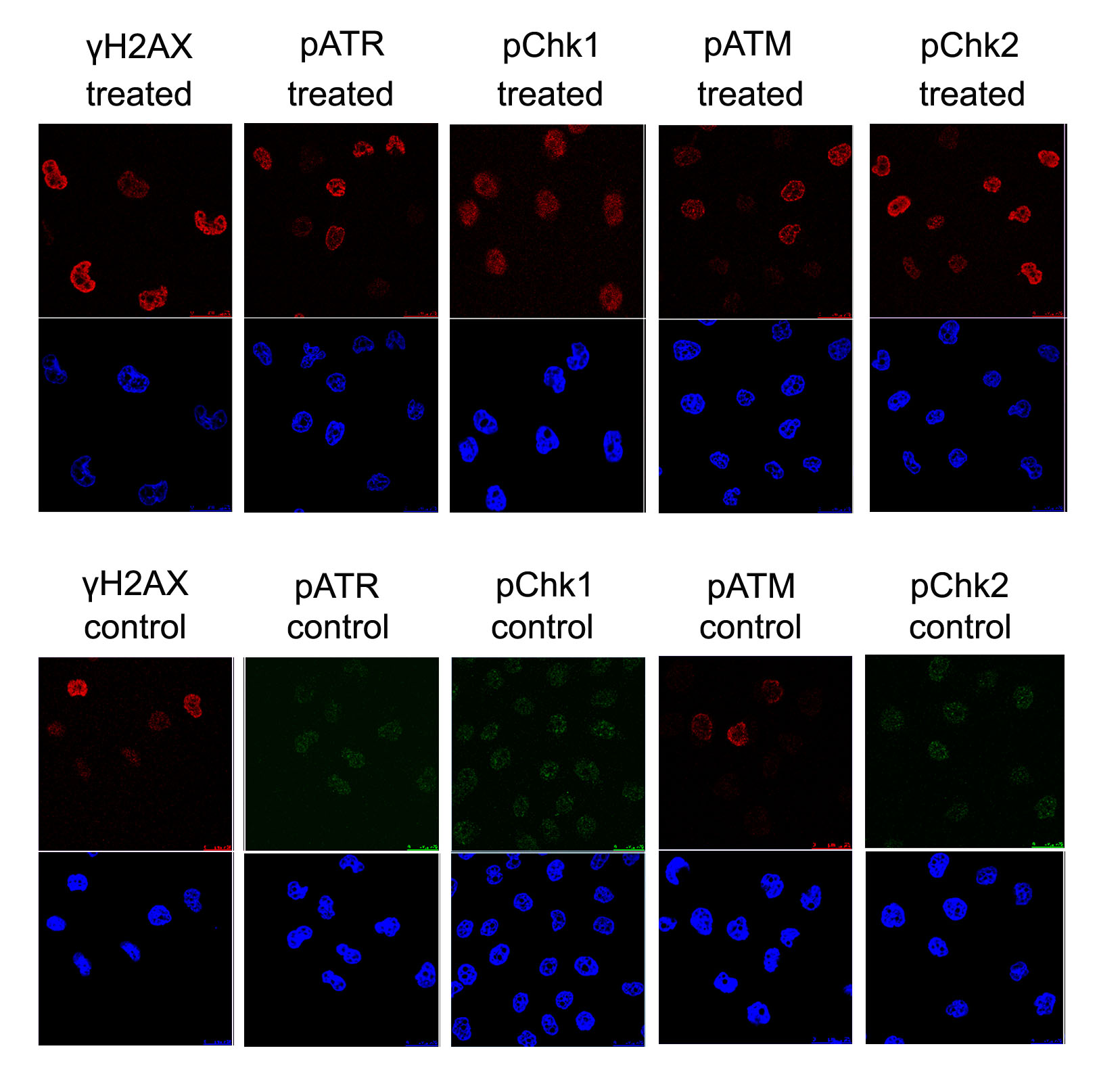

Supplement: S1 Fig — (A2780 data corresponding to results shown in Fig 1D) Typical images showing the key molecules under study using microscope analysis of A2780 cells treated with 100 μg/ml cisplatin for 3h; upper images, immunofluorescence antigen staining; bottom images, cell nuclei labeled with DAPI. The key molecules of control/non-treated A2780 cells are also presented. (JPG) [file pone.0256051.s001.jpg]

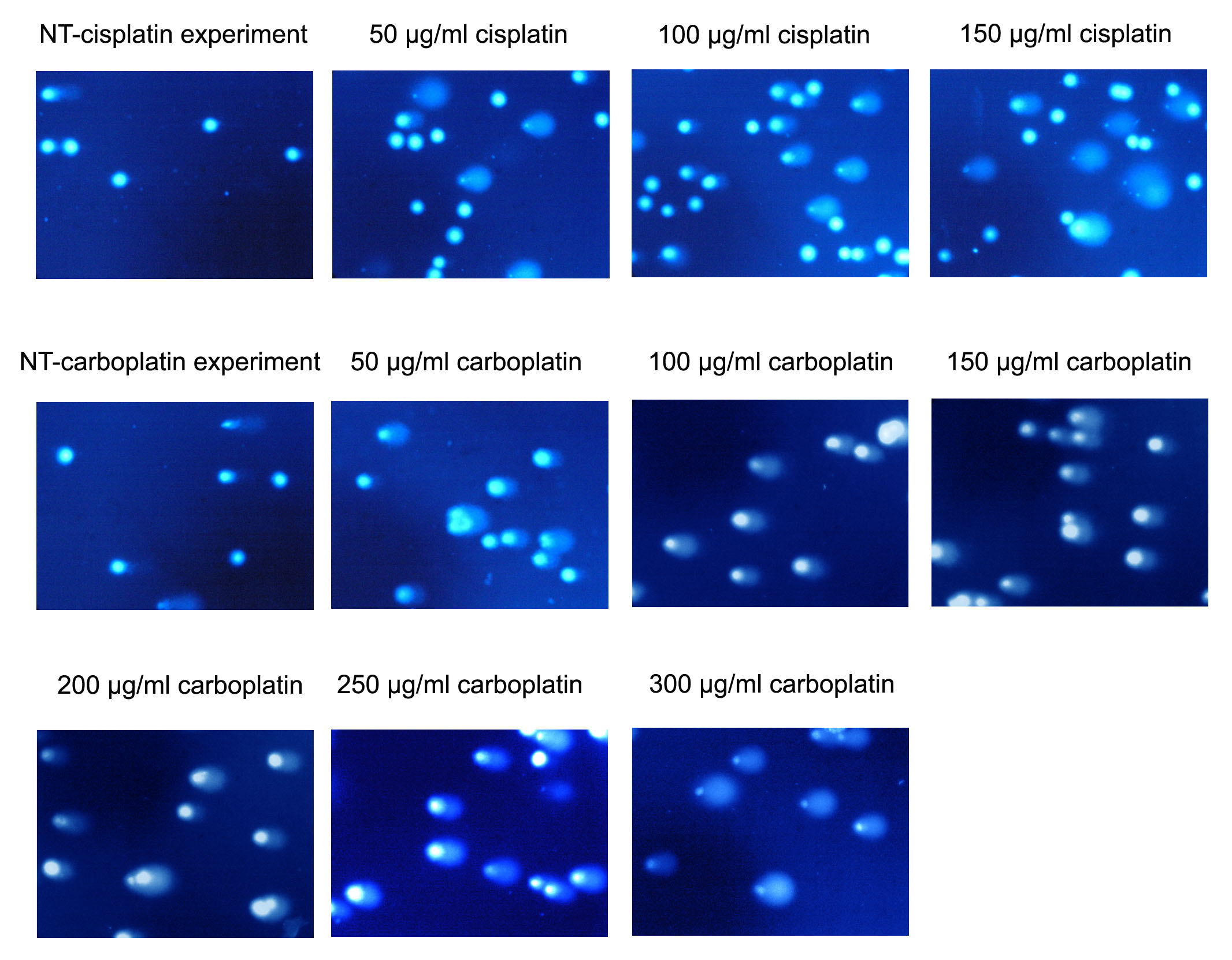

Supplement: S2 Fig — (A2780 data corresponding to results shown in Fig 2C) Typical comet assay images of A2780 cells treated with cisplatin (0–150 μg/ml) for 3h or carboplatin (0–300 μg/ml) for 24h and analyzed at the end of the treatment. NT, non-treated. (JPG) [file pone.0256051.s002.jpg]

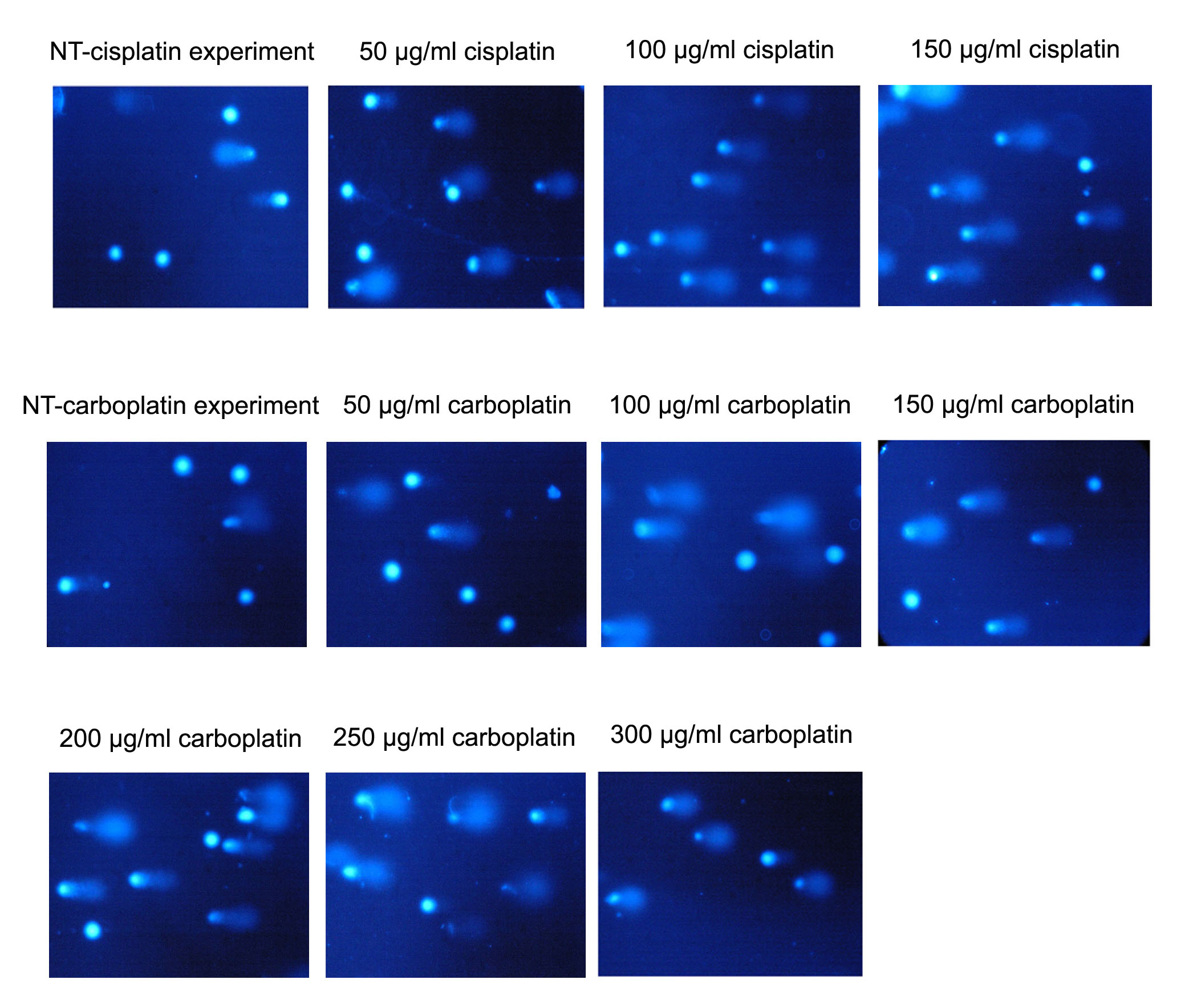

Supplement: S3 Fig — (A2780/C30 data corresponding to results shown in Fig 2C) Typical comet assay images of A2780/C30 cells treated with cisplatin (0–150 μg/ml) for 3h or carboplatin (0–300 μg/ml) for 24h and analyzed at the end of the treatment. NT, non-treated. (JPG) [file pone.0256051.s003.jpg]

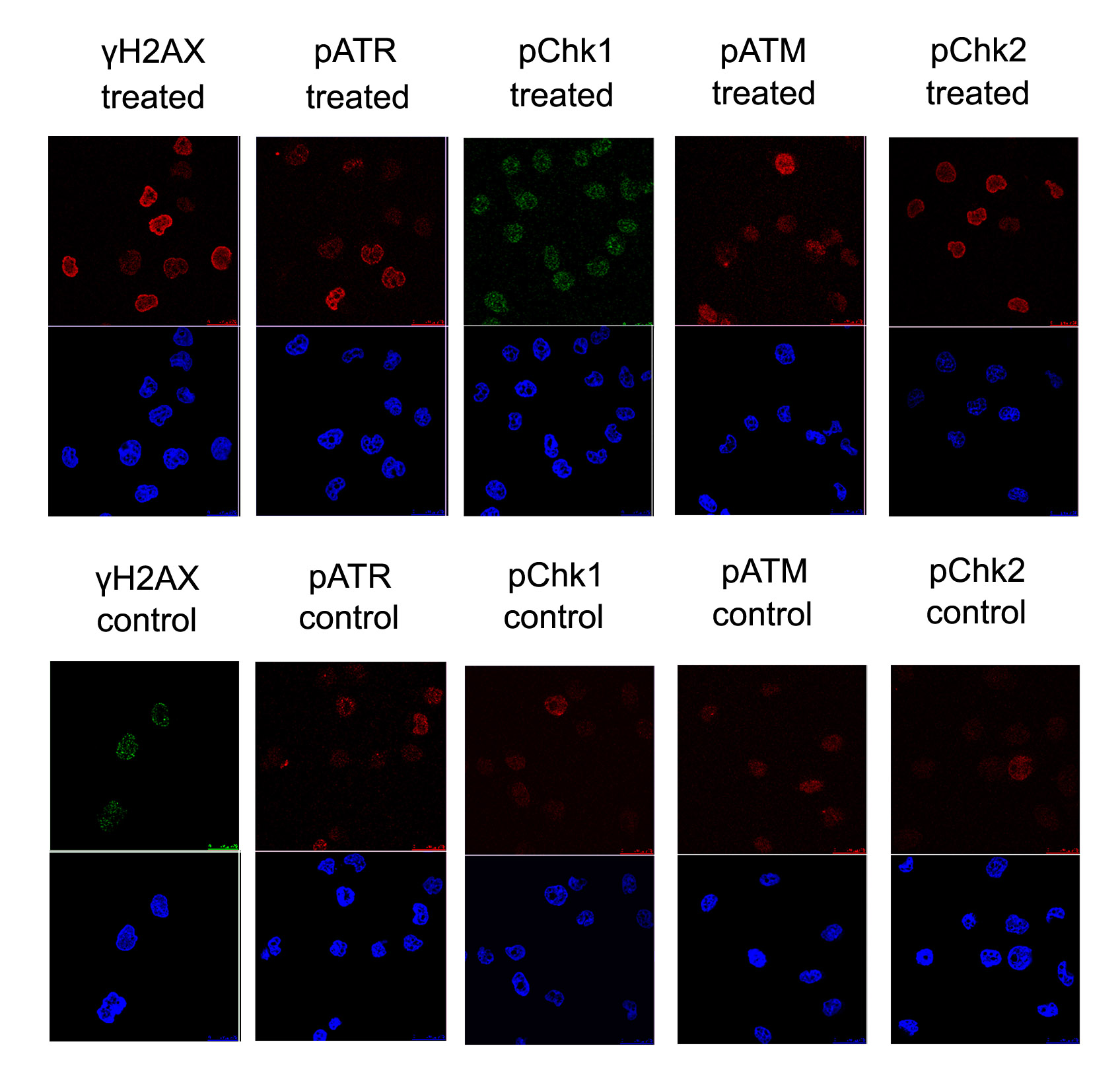

Supplement: S6 File — (ZIP) [file pone.0256051.s006.zip › S6 FILE/Figure Q-6a-1.jpg]

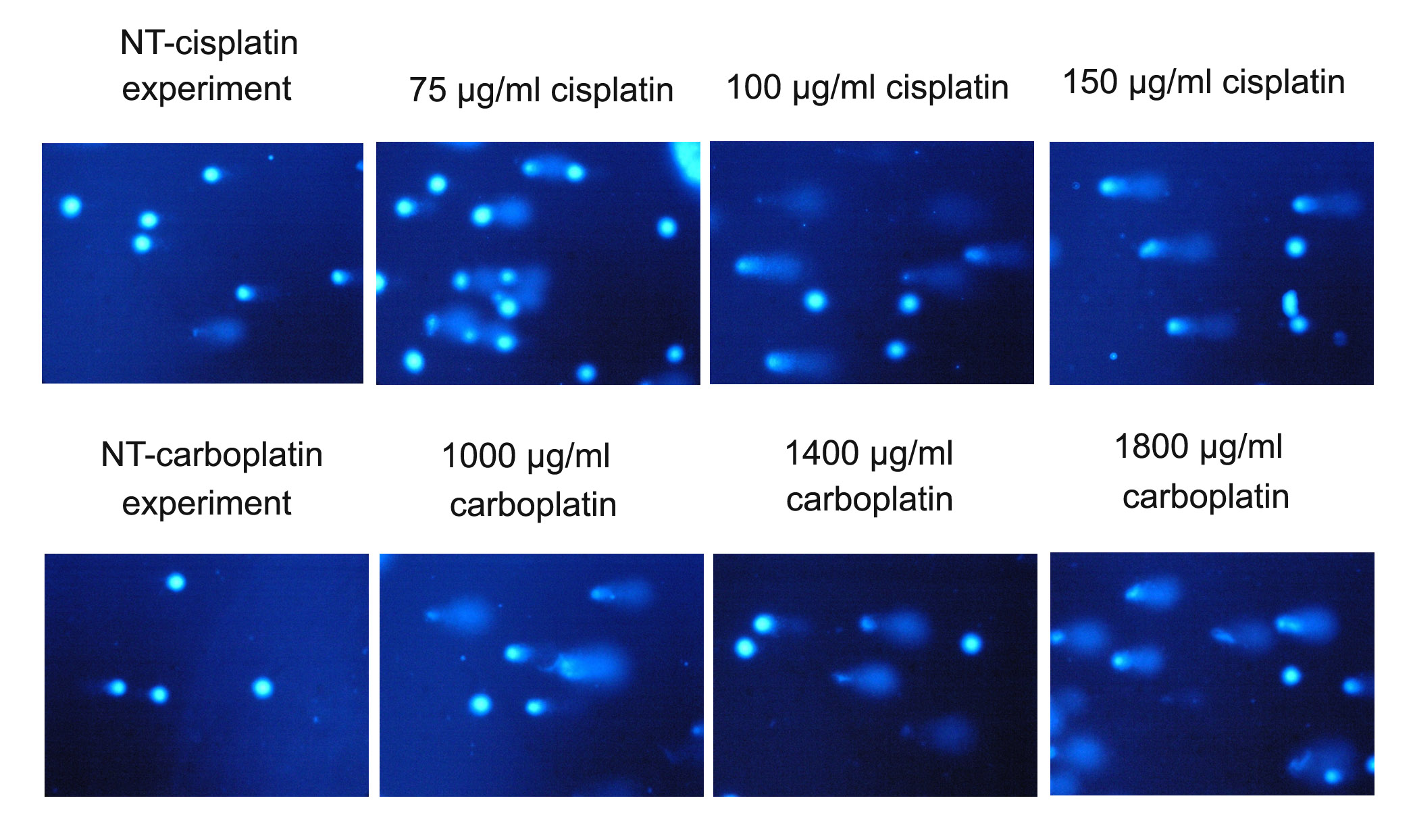

Supplement: S6 File — (ZIP) [file pone.0256051.s006.zip › S6 FILE/Figure Q-6a-2.jpg]

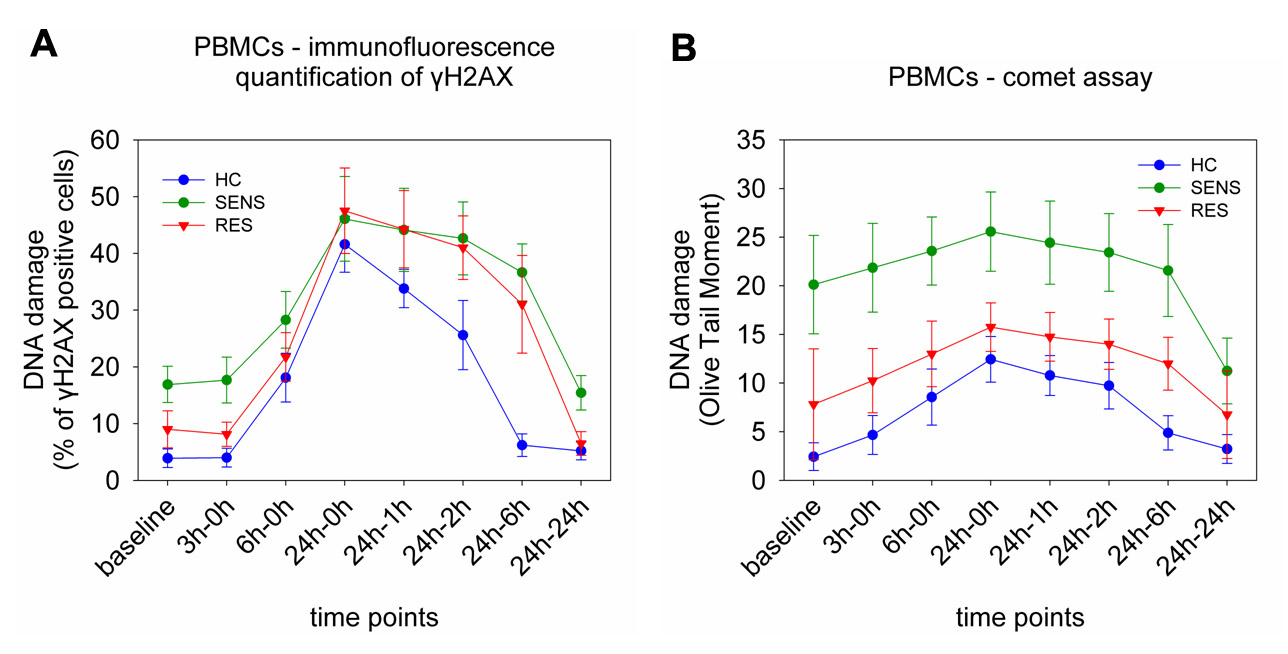

Supplement: S7 File — (ZIP) [file pone.0256051.s007.zip › S7 FILE/Figure Q-7a-1.jpg]

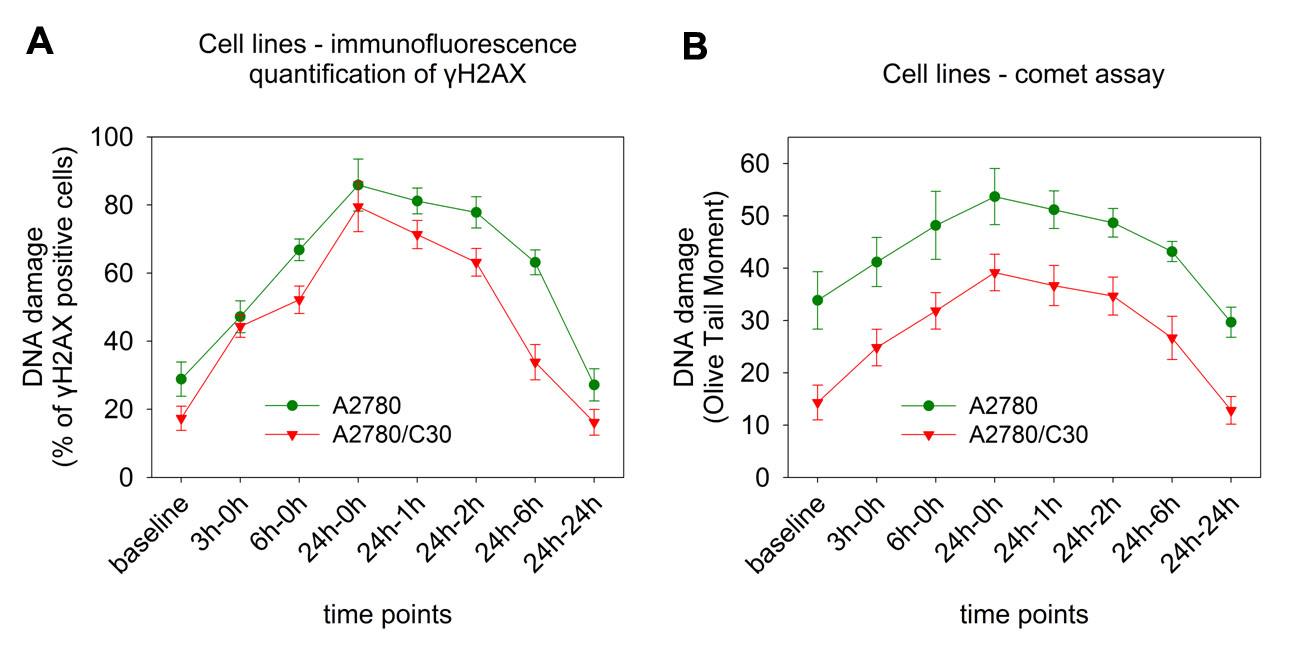

Supplement: S7 File — (ZIP) [file pone.0256051.s007.zip › S7 FILE/Figure Q-7c-1.jpg]

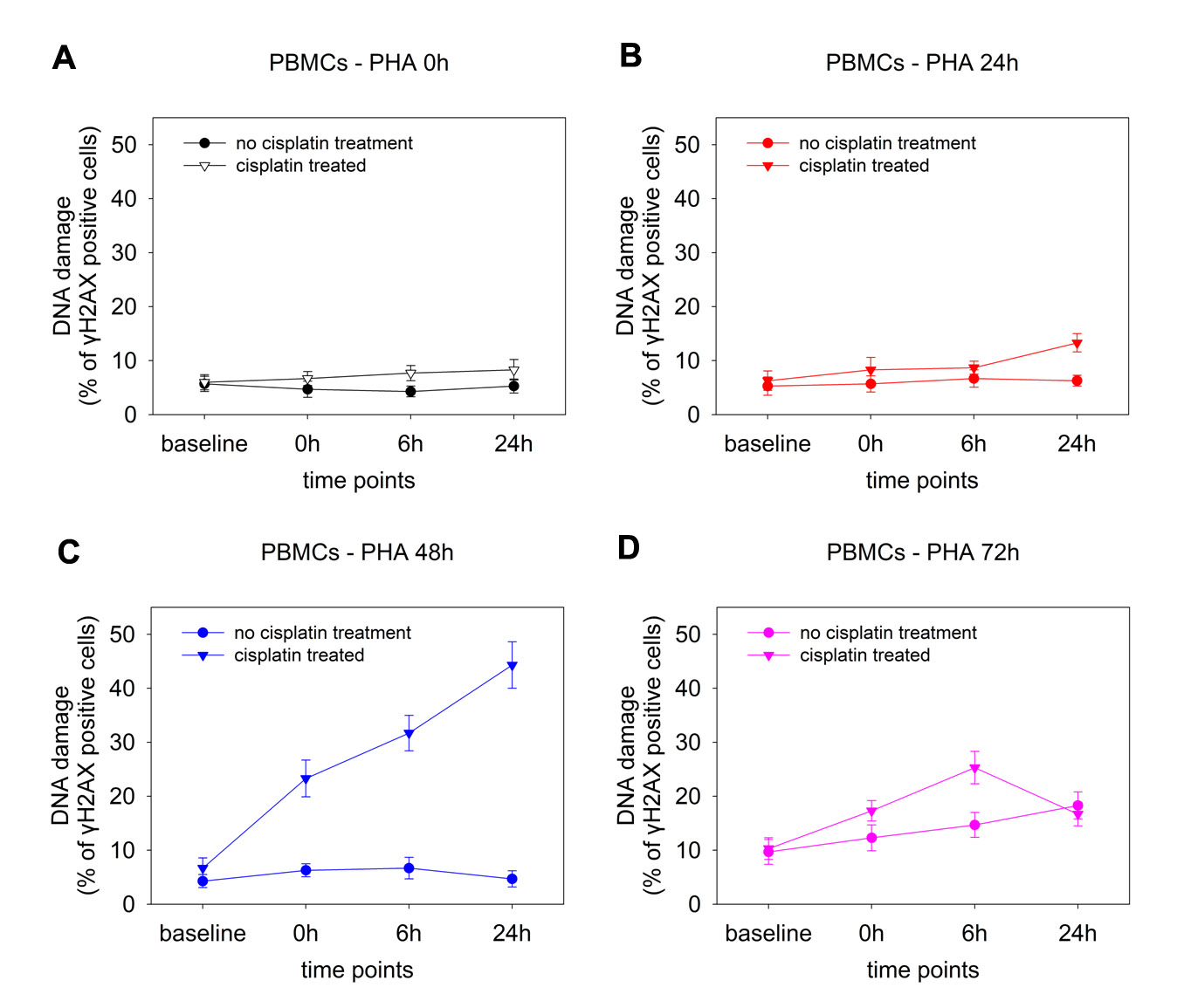

Supplement: S7 File — (ZIP) [file pone.0256051.s007.zip › S7 FILE/Figure Q-7d-1.jpg]
