# Supplementary material for: Correction: Aberrant DNA Damage Response Pathways May Predict the Outcome of Platinum Chemotherapy in Ovarian Cancer
Source: PLoS One. 2021 Aug 5;16(8):e0256051. doi: 10.1371/journal.pone.0256051 (PMC8341518; doi:10.1371/journal.pone.0256051)
Supplement: S4 File — (DOC) [file pone.0256051.s004.doc]

**S4 File. Tables Q-4-1 and Q-4-2: Control data corresponding to results shown in Figure 3B and 3D**

Control data showing expression levels throughout the time course in non-treated PBMCs are presented in Table Q-4-1 (Figure 3B) and Table Q-4-2 (Figure 3D).

| **Table Q-4-1:** PBMCs from nine healthy volunteers were incubated in drug-free medium for various time-periods and analyzed using confocal microscopy | | | | | | | | | | |
| --- | --- | --- | --- | --- | --- | --- | --- | --- | --- | --- |
| Post-incubation time (h) | % positive cells | | | | | | | | | |
|  | pATR | | pATM | | pCHK1 | | pCHK2 | | γH2AX | |
|  | mv | sd | mv | sd | mv | sd | mv | sd | mv | sd |
| 0/0* | 2.0 | 1.2 | 4.5 | 1.1 | 5.0 | 1.2 | 4.0 | 0.7 | 3.9 | 0.9 |
| 0 | 4.3 | 2.5 | 5.3 | 2.1 | 6.7 | 1.2 | 4.7 | 2.1 | 5.3 | 2.1 |
| 6 | 4.1 | 0.9 | 4.0 | 0.8 | 4.6 | 1.5 | 5.5 | 1.1 | 4.5 | 0.8 |
| 24 | 3.2 | 1.0 | 5.2 | 1.2 | 5.5 | 1.1 | 4.4 | 0.8 | 4.9 | 1.3 |
| *baseline | | | | | | | | | | |

| **Table Q-4-2:** PBMCs from nine healthy volunteers were incubated in drug-free medium for various time-periods and analyzed using confocal microscopy | | | | | | | | | | |
| --- | --- | --- | --- | --- | --- | --- | --- | --- | --- | --- |
| Incubation time (h) | % positive cells | | | | | | | | | |
|  | pATR | | pATM | | pCHK1 | | pCHK2 | | γH2AX | |
|  | mv | sd | mv | sd | mv | sd | mv | sd | mv | sd |
| 0 | 3.0 | 1.1 | 5.0 | 1.2 | 4.0 | 1.4 | 5.0 | 0.9 | 4.0 | 1.6 |
| 3 | 4.4 | 0.8 | 4.7 | 0.7 | 5.1 | 1.2 | 5.4 | 0.9 | 4.9 | 1.1 |
| 6 | 3.6 | 1.0 | 4.4 | 0.9 | 5.4 | 0.8 | 5.9 | 1.0 | 5.1 | 1.4 |
| 24 | 4.1 | 1.4 | 5.7 | 1.1 | 4.6 | 0.9 | 4.4 | 1.2 | 5.4 | 1.0 |
