# Supplementary material for: Correction: Aberrant DNA Damage Response Pathways May Predict the Outcome of Platinum Chemotherapy in Ovarian Cancer
Source: PLoS One. 2021 Aug 5;16(8):e0256051. doi: 10.1371/journal.pone.0256051 (PMC8341518; doi:10.1371/journal.pone.0256051)
Supplement: S5 File — (DOC) [file pone.0256051.s005.doc]

**S5 File. Tables Q-5a-1, Q-5a-2, Q-5b-1, Q-5b-2, Q-5c-1, Q-5c-2, Q-5d-1: Results for serous tumors and clear ovarian cancers**

Q-5a. “in serous tumors only, using γH2AX staining, sensitive patients (n = 6) showed higher levels of intrinsic DNA damage (mean value, 16.6% positive cells; range, 13.1–23.2%) than resistant patient (n = 1; 7.5%). In addition, in clear cell ovarian cancer only, sensitive patient (n = 1) exhibited 18.5% positive cells, while resistant patients (n = 3) only 9.4% (range, 6.3–13.7%). Similar results were obtained using comet assay.”

Tables Q-5a-1 and Q-5a-2 below show the results that support our conclusions. Also, see the “*Minimal dataset Stefanou et al PLOS ONE 2014*” excel file, pages 14 and 15 (Helios Digital Repository).

| **Table Q-5a-1:** Intrinsic DNA damage in PBMCs from healthy controls and OC patients using immunofluorescence quantification of γH2AX | | | |
| --- | --- | --- | --- |
| # | % of γH2AX positive cells | | |
|  | Healthy volunteers | Sensitive patients | Resistant patients |
| 1 | 2.1 | 18.5* | 6.3 |
| 2 | 2.2 | 13.1 | 8.5 |
| 3 | 2.5 | 15.7 | 13.7 |
| 4 | 2.8 | 15.7 | 7.5 |
| 5 | 3.8 | 16.0 |  |
| 6 | 4.0 | 16.1 |  |
| 7 | 5.0 | 23.2 |  |
| 8 | 6.0 |  |  |
| 9 | 6.5 |  |  |
| mv | 3.9 | 16.9 | 9.0 |
| max | 6.5 | 23.2 | 13.7 |
| min | 2.1 | 13.1 | 6.3 |
| *clear cells | | | |

Similar results were obtained using comet assay.

| **Table Q-5a-2:** Intrinsic DNA damage in PBMCs from healthy controls and OC patients using alkaline comet assay | | | |
| --- | --- | --- | --- |
| # | Olive Tail Moment (OTM) | | |
|  | Healthy volunteers | Sensitive patients | Resistant patients |
| 1 | 1.1 | 17.0* | 3.7 |
| 2 | 1.3 | 16.7 | 7.0 |
| 3 | 1.6 | 17.5 | 16.1 |
| 4 | 1.7 | 18.5 | 4.5 |
| 5 | 2.2 | 19.0 |  |
| 6 | 2.3 | 21.0 |  |
| 7 | 2.9 | 31.1 |  |
| 8 | 3.0 |  |  |
| 9 | 5.8 |  |  |
| mv | 2.4 | 20.1 | 7.8 |
| max | 5.8 | 31.1 | 16.1 |
| min | 1.1 | 16.7 | 3.7 |
| *clear cells | | | |

Q-5b. “in serous tumors only, the γH2AX foci were removed with t1/2 = 15.4h in sensitive patients (n = 6; range, 12.8–18.2h) and t1/2 = 8.4h in the resistant one (n = 1). In addition, in clear cell ovarian cancer only, sensitive patient (n = 1) showed t1/2 = 17.1h, while resistant patients t1/2 = 8.9h (n = 3; range, 6.1–10.6h). Similar results were obtained using comet assay.”

Tables Q-5b-1 and Q-5b-2 below show the results that support our conclusions. Also, see the “*Additional data*” excel file, pages 14 and 15 (Helios Digital Repository).

| **Table Q-5b-1:** t1/2 in h for γH2AX foci removal using confocal microscopy | | | |
| --- | --- | --- | --- |
| # | t1/2 (h) | | |
|  | Healthy volunteers | Sensitive patients | Resistant patients |
| 1 | 1.5 | 17.1* | 6.1 |
| 2 | 2.8 | 12.8 | 10.1 |
| 3 | 2.7 | 13.4 | 10.6 |
| 4 | 2.9 | 14.5 | 8.4 |
| 5 | 2.4 | 15.2 |  |
| 6 | 2.9 | 17.5 |  |
| 7 | 2.7 | 18.2 |  |
| 8 | 2.9 |  |  |
| 9 | 3.2 |  |  |
| mv | 2.7 | 15.5 | 8.8 |
| max | 3.2 | 18.2 | 10.6 |
| min | 1.5 | 12.8 | 6.1 |
| *clear cells | | | |

Similar results were obtained using comet assay.

| **Table Q-5b-2:** t1/2 in h for DNA damage repair using comet assay | | | |
| --- | --- | --- | --- |
| # | t1/2 (h) | | |
|  | Healthy volunteers | Sensitive patients | Resistant patients |
| 1 | 4.5 | 19.7* | 8.5 |
| 2 | 2.3 | 17.3 | 13.9 |
| 3 | 5.0 | 21.0 | 15.2 |
| 4 | 6.0 | 21.2 | 14.1 |
| 5 | 4.3 | 21.8 |  |
| 6 | 4.7 | 23.0 |  |
| 7 | 4.7 | 26.0 |  |
| 8 | 6.7 |  |  |
| 9 | 5.3 |  |  |
| mv | 4.8 | 21.4 | 12.9 |
| max | 6.7 | 26.0 | 15.2 |
| min | 2.3 | 17.3 | 8.5 |
| *clear cells | | | |

Q-5c. “in serous tumors only, sensitive patients showed AUC values of 28400 (n = 6; range, 25100–34400), while the platinum-resistant patient 15200 (n = 1). In addition, in clear cell ovarian cancer only, sensitive patient (n = 1) exhibited AUC values of 29000, while resistant patients (n = 3) only 18500 (range, 13700–23500). Similar results were obtained using comet assay.”

Tables Q-5c-1 and Q-5c-2 below show the results that support our conclusions. Also, see the “*Minimal dataset Stefanou et al PLOS ONE 2014*” excel file, pages 16 and 17 (Helios Digital Repository).

| **Table Q-5c-1:** Carboplatin-induced DNA damage (expressed as AUC; immunofluorescence quantification of γH2AX) in PBMCs from healthy volunteers and OC patients | | | |
| --- | --- | --- | --- |
| # | AUC [(% of γH2AX positive cells) x (carboplatin dose)] | | |
|  | Healthy volunteers | Sensitive patients | Resistant patients |
| 1 | 18700 | 29000* | 23500 |
| 2 | 13060 | 34400 | 19300 |
| 3 | 11250 | 31200 | 13700 |
| 4 | 10000 | 27400 | 15200 |
| 5 | 8510 | 26550 |  |
| 6 | 8100 | 25850 |  |
| 7 | 8010 | 25100 |  |
| 8 | 7570 |  |  |
| 9 | 7500 |  |  |
| mv | 10300 | 28500 | 17925 |
| max | 18700 | 34400 | 23500 |
| min | 7500 | 25100 | 13700 |
| *clear cells | | | |

Similar results were obtained using comet assay.

| **Table Q-5c-2:** Carboplatin-induced DNA damage (expressed as AUC; alkaline comet assay) in PBMCs from healthy volunteers and OC patients | | | |
| --- | --- | --- | --- |
| # | AUC (OTM x carboplatin dose) | | |
|  | Healthy volunteers | Sensitive patients | Resistant patients |
| 1 | 14930 | 26270* | 21870 |
| 2 | 14645 | 29640 | 20310 |
| 3 | 13343 | 28350 | 6840 |
| 4 | 12060 | 22889 | 15940 |
| 5 | 11700 | 19257 |  |
| 6 | 8755 | 17500 |  |
| 7 | 6669 | 14990 |  |
| 8 | 5500 |  |  |
| 9 | 1050 |  |  |
| mv | 9850 | 22699 | 16240 |
| max | 14930 | 29640 | 21870 |
| min | 1050 | 14990 | 6840 |
| *clear cells | | | |

Q-5d. “in serous tumors only, sensitive patients (n = 6) showed evidence of apoptosis at carboplatin doses of 636μg/ml (range, 390–1050μg/ml) and the platinum-resistant patient at 1370μg/ml (n = 1). In addition, in clear cell ovarian cancer only, the corresponding values were 420μg/ml for the sensitive patient (n = 1) and 877μg/ml (n = 3; range, 800–940μg/ml) for the resistant ones.”

Table Q-5d-1 below shows the results that support our conclusions. Also, see the “*Minimal dataset Stefanou et al PLOS ONE 2014*” excel file, page 18 (Helios Digital Repository).

| **Table Q-5d-1:** Apoptosis rates, expressed as doses of carboplatin inducing apoptosis, in the three groups of individuals | | | |
| --- | --- | --- | --- |
| # | Carboplatin dose inducing apoptosis (μg/ml) | | |
|  | Healthy volunteers | Sensitive patients | Resistant patients |
| 1 | 1320 | 420* | 800 |
| 2 | 1350 | 390 | 890 |
| 3 | 1400 | 425 | 940 |
| 4 | 1400 | 550 | 1370 |
| 5 | 1600 | 600 |  |
| 6 | 1700 | 800 |  |
| 7 | 1800 | 1050 |  |
| 8 | 1800 |  |  |
| 9 | 2000 |  |  |
| mv | 1597 | 605.0 | 1000.0 |
| max | 2000 | 1050.0 | 1370.0 |
| min | 1320 | 390.0 | 800.0 |
| *clear cells | | | |
