# Supplementary material for: Correction: Aberrant DNA Damage Response Pathways May Predict the Outcome of Platinum Chemotherapy in Ovarian Cancer
Source: PLoS One. 2021 Aug 5;16(8):e0256051. doi: 10.1371/journal.pone.0256051 (PMC8341518; doi:10.1371/journal.pone.0256051)
Supplement: S6 File — (ZIP) [file pone.0256051.s006.zip › S6 FILE/S6 File.doc]

**S6 File. Figures Q-6a-1, Q-6a-2 and Table Q-6c-1: Supporting data for the results shown in Figures 3, 4E, 4F and 5A-C**

Q-6a. Representative image data demonstrating the results shown in Figure 3.

Data are presented in the “*Minimal dataset Stefanou et al PLOS ONE 2014*” excel file (Helios Digital Repository), pages 8-13. Please, also see Figures Q-6a-1 and Q-6a-2 below.

**Figure Q-6a-1: Changes in key molecules of the DDR pathways in PBMCs from healthy volunteers.** Typical images showing the key molecules under study using microscope analysis of PBMCs from healthy volunteers treated with 1400 μg/ml carboplatin for 24h; upper images, immunofluorescence antigen staining; bottom images, cell nuclei labeled with DAPI. The key molecules of control/non-treated PBMCs are also presented.

**Figure Q-6a-2: Measurement of DNA damage in PBMCs from healthy volunteers using alkaline comet assay.** Typical comet assay images of PBMCs from healthy volunteers treated with cisplatin (0-150 μg/ml) for 3h or with carboplatin (0-1800 μg/ml) for 24h and analyzed using alkaline comet assay. NT, non-treated.

Q-6b. Supporting data for the results shown in Figure 4E, 4F.

Please see Tables Q-5c-1 and Q-5c-2 (Supporting Information, S5 File). Also, see the “*Minimal dataset Stefanou et al PLOS ONE 2014*” excel file, pages 16 and 17 (Helios Digital Repository).

Q-6c. Supporting data for the results shown in Figure 5A-C.

Please see Tables Q-5c-1, Q-5c-2, Q-5d-1 (Supporting Information, S5 File) and Q-6c-1 (below). Also, see the “*Minimal dataset Stefanou et al PLOS ONE 2014*” excel file, pages 16-18, and the “*Additional data*” excel file, page 16 (Helios Digital Repository).

| **Table Q-6c-1:** Apoptosis rates (ELISA assay) and DNA damage (expressed as AUC) in PBMCs from the same individuals | | | |
| --- | --- | --- | --- |
|  | AUC [(% of γH2AX positive cells) x (carboplatin dose)] | AUC (OTM x carboplatin dose) | Carboplatin dose inducing apoptosis (μg/ml) |
| Healthy volunteers | 18700 | 14930 | 1320 |
|  | 13060 | 14645 | 1350 |
|  | 11250 | 13343 | 1400 |
|  | 10000 | 12060 | 1400 |
|  | 8510 | 11700 | 1600 |
|  | 8100 | 8755 | 1700 |
|  | 8010 | 6669 | 1800 |
|  | 7570 | 5500 | 1800 |
|  | 7500 | 1050 | 2000 |
| Sensitive patients | 29000* | 26270 | 420 |
|  | 34400 | 29640 | 390 |
|  | 31200 | 28350 | 425 |
|  | 27400 | 22889 | 550 |
|  | 26550 | 19257 | 600 |
|  | 25850 | 17500 | 800 |
|  | 25100 | 14990 | 1050 |
| Resistant patients | 23500 | 21870 | 800 |
|  | 19300 | 20310 | 890 |
|  | 13700 | 6840 | 940 |
|  | 15200 | 15940 | 1370 |
| *clear cells | | | |
