# Supplementary material for: Correction: Aberrant DNA Damage Response Pathways May Predict the Outcome of Platinum Chemotherapy in Ovarian Cancer
Source: PLoS One. 2021 Aug 5;16(8):e0256051. doi: 10.1371/journal.pone.0256051 (PMC8341518; doi:10.1371/journal.pone.0256051)
Supplement: S7 File — (ZIP) [file pone.0256051.s007.zip › S7 FILE/S7 File.doc]

**S7 File. Tables Q-7a-1, Q-7a-2, Q-7a-3, Q-7a-4, Q-7a-5, Q-7a-6, Q-7c-1, Q-7c-2, Q-7c-3, Q-7c-4, Q-7d-1, Q-7d-2, Q-7d-3, Q-7d-4 and Figures Q-7a-1, Q-7c-1, Q-7d-1: Results to support statements for which data were not shown in the original article**

Q-7a. “Both γH2AX immunofluorescence staining and comet assay showed similar results.”

Tables Q-7a-1, Q-7a-2, Q-7a-3, Q-7a-4, Q-7a-5, Q-7a-6 and Figure Q-7a-1 (below) show the data that support our conclusion. Also, see the “*Additional data*” excel file, pages 17-22 (Helios Digital Repository). The results presented in Tables Q-5a-1, Q-5a-2, Q-5b-1, Q-5b-2, Q-5c-1, Q-5c-2 (Supporting Information, S5 File), as well as in Tables Q-7c-1, Q-7c-2, Q-7c-3, Q-7c-4 and Figure Q-7c-1 (see below) also support the conclusion that “both γH2AX immunofluorescence staining and comet assay showed similar results”.

| **Table Q-7a-1:** PBMCs from healthy volunteers were treated with 1400 μg/ml carboplatin for 0, 3, 6 or 24h, post-incubated in drug-free medium for 0, 1, 2, 6 or 24h and the drug-induced DNA damage was analyzed using immunofluorescence quantification of γH2AX | | | | | | | | |
| --- | --- | --- | --- | --- | --- | --- | --- | --- |
| # | % of γH2AX positive cells | | | | | | | |
|  | 0h treatment  (intrinsic) | 3h treatment | 6h treatment | 24h treatment | | | | |
|  |  |  |  | Post-incubation time | | | | |
|  |  |  |  | 0h | 1h | 2h | 6h | 24h |
| 1 | 2.1 | 2 | 12 | 35 | 29 | 12 | 3 | 3 |
| 2 | 2.2 | 2 | 15 | 36 | 30 | 23 | 4 | 4 |
| 3 | 2.5 | 3 | 13 | 38 | 30 | 24 | 5 | 4 |
| 4 | 2.8 | 3 | 17 | 38 | 34 | 25 | 6 | 5 |
| 5 | 3.8 | 4 | 21 | 43 | 34 | 25 | 6 | 5 |
| 6 | 4.0 | 5 | 22 | 43 | 35 | 28 | 7 | 5 |
| 7 | 5.0 | 5 | 19 | 46 | 37 | 29 | 8 | 6 |
| 8 | 6.0 | 5 | 25 | 47 | 37 | 31 | 8 | 7 |
| 9 | 6.5 | 7 | 19 | 48 | 38 | 33 | 9 | 8 |
| mv | 3.9 | 4.0 | 18.1 | 41.6 | 33.8 | 25.6 | 6.2 | 5.2 |
| max | 6.5 | 7.0 | 25.0 | 48.0 | 38.0 | 33.0 | 9.0 | 8.0 |
| min | 2.1 | 2.0 | 12.0 | 35.0 | 29.0 | 12.0 | 3.0 | 3.0 |

| **Table Q-7a-2:** PBMCs from sensitive patients were treated with 1400 μg/ml carboplatin for 0, 3, 6 or 24h, post-incubated in drug-free medium for 0, 1, 2, 6 or 24h and the drug-induced DNA damage was analyzed using immunofluorescence quantification of γH2AX | | | | | | | | |
| --- | --- | --- | --- | --- | --- | --- | --- | --- |
| # | % of γH2AX positive cells | | | | | | | |
|  | 0h treatment  (intrinsic) | 3h treatment | 6h treatment | 24h treatment | | | | |
|  |  |  |  | Post-incubation time | | | | |
|  |  |  |  | 0h | 1h | 2h | 6h | 24h |
| 1* | 18.5 | 17.8 | 30 | 50 | 47 | 47 | 40 | 14 |
| 2 | 13.1 | 12.4 | 20 | 35 | 34 | 32 | 29.0 | 11 |
| 3 | 15.7 | 15.2 | 24 | 39 | 37 | 37 | 32.6 | 13 |
| 4 | 15.7 | 16.2 | 27 | 42 | 40 | 41 | 33.5 | 15 |
| 5 | 16.0 | 17.4 | 30 | 49 | 46 | 44 | 38.0 | 17 |
| 6 | 16.1 | 19.3 | 33 | 54 | 52 | 48 | 41.3 | 19 |
| 7 | 23.2 | 25.4 | 34 | 54 | 53 | 50 | 42.5 | 19 |
| mv | 16.9 | 17.7 | 28.3 | 46.1 | 44.1 | 42.6 | 36.6 | 15.4 |
| max | 23.2 | 25.4 | 34.0 | 54.0 | 53.0 | 50.0 | 42.5 | 19.0 |
| min | 13.1 | 12.4 | 20.0 | 35.0 | 34.0 | 32.0 | 29.0 | 11.0 |
| *clear cells | | | | | | | | |

| **Table Q-7a-3:** PBMCs from resistant patients were treated with 1400 μg/ml carboplatin for 0, 3, 6 or 24h, post-incubated in drug-free medium for 0, 1, 2, 6 or 24h and the drug-induced DNA damage was analyzed using immunofluorescence quantification of γH2AX | | | | | | | | |
| --- | --- | --- | --- | --- | --- | --- | --- | --- |
| # | % of γH2AX positive cells | | | | | | | |
|  | 0h treatment  (intrinsic) | 3h treatment | 6h treatment | 24h treatment | | | | |
|  |  |  |  | Post-incubation time | | | | |
|  |  |  |  | 0h | 1h | 2h | 6h | 24h |
| 1* | 6.3 | 5.9 | 17 | 39 | 35 | 33 | 20 | 4 |
| 2 | 8.5 | 8.1 | 23 | 49 | 47 | 43 | 35 | 7 |
| 3 | 13.7 | 11 | 27 | 57 | 51 | 46 | 40 | 9 |
| 4 | 7.5 | 7.5 | 20 | 45 | 44 | 42 | 29 | 6 |
| mv | 9.0 | 8.1 | 21.8 | 47.5 | 44.3 | 41.0 | 31.0 | 6.5 |
| max | 13.7 | 11.0 | 27.0 | 57.0 | 51.0 | 46.0 | 40.0 | 9.0 |
| min | 6.3 | 5.9 | 17.0 | 39.0 | 35.0 | 33.0 | 20.0 | 4.0 |
| *clear cells | | | | | | | | |

Similar results were obtained using comet assay.

| **Table Q-7a-4:** PBMCs from healthy volunteers were treated with 1400 μg/ml carboplatin for 0, 3, 6 or 24h, post-incubated in drug-free medium for 0, 1, 2, 6 or 24h and the drug-induced DNA damage was analyzed using comet assay | | | | | | | | |
| --- | --- | --- | --- | --- | --- | --- | --- | --- |
| # | Olive Tail Moment (OTM) | | | | | | | |
|  | 0h treatment  (intrinsic) | 3h treatment | 6h treatment | 24h treatment | | | | |
|  |  |  |  | Post-incubation time | | | | |
|  |  |  |  | 0h | 1h | 2h | 6h | 24h |
| 1 | 1.1 | 2 | 5 | 9 | 8 | 7 | 3 | 2 |
| 2 | 1.3 | 3 | 6 | 10 | 9 | 6 | 3 | 2 |
| 3 | 1.6 | 3 | 6 | 10 | 9 | 8 | 4 | 2 |
| 4 | 1.7 | 4 | 7 | 12 | 10 | 10 | 4 | 2 |
| 5 | 2.2 | 4 | 8 | 13 | 10 | 10 | 4 | 3 |
| 6 | 2.3 | 5 | 9 | 14 | 12 | 11 | 5 | 3 |
| 7 | 2.9 | 6 | 11 | 14 | 12 | 11 | 6 | 4 |
| 8 | 3.0 | 7 | 12 | 14 | 13 | 12 | 7 | 5 |
| 9 | 5.8 | 8 | 13 | 16 | 14 | 13 | 8 | 6 |
| mv | 2.4 | 4.7 | 8.6 | 12.4 | 10.8 | 9.7 | 4.9 | 3.2 |
| max | 5.8 | 8.0 | 13.0 | 16.0 | 14.0 | 13.0 | 8.0 | 6.0 |
| min | 1.1 | 2.0 | 5.0 | 9.0 | 8.0 | 5.7 | 3.0 | 2.0 |

| **Table Q-7a-5:** PBMCs from sensitive patients were treated with 1400 μg/ml carboplatin for 0, 3, 6 or 24h, post-incubated in drug-free medium for 0, 1, 2, 6 or 24h and the drug-induced DNA damage was analyzed using comet assay | | | | | | | | |
| --- | --- | --- | --- | --- | --- | --- | --- | --- |
| # | Olive Tail Moment (OTM) | | | | | | | |
|  | 0h treatment  (intrinsic) | 3h treatment | 6h treatment | 24h treatment | | | | |
|  |  |  |  | Post-incubation time | | | | |
|  |  |  |  | 0h | 1h | 2h | 6h | 24h |
| 1* | 17.0 | 18 | 21 | 23 | 22 | 20 | 16 | 9.0 |
| 2 | 16.7 | 15 | 18 | 21 | 19 | 18 | 15 | 6.5 |
| 3 | 17.5 | 20 | 22 | 23 | 22 | 22 | 21 | 10.0 |
| 4 | 18.5 | 22 | 24 | 23 | 22 | 22 | 22 | 11.0 |
| 5 | 19.0 | 24 | 25 | 28 | 27 | 26 | 24 | 12.0 |
| 6 | 21.0 | 26 | 27 | 29 | 28 | 27 | 25 | 13.0 |
| 7 | 31.1 | 28 | 28 | 32 | 31 | 29 | 28 | 17.2 |
| mv | 20.1 | 21.9 | 23.6 | 25.6 | 24.4 | 23.4 | 21.6 | 11.2 |
| max | 31.1 | 28.0 | 28.0 | 32.0 | 31.0 | 29.0 | 28.0 | 17.2 |
| min | 16.7 | 15.0 | 18.0 | 21.0 | 19.0 | 18.0 | 15.0 | 6.5 |
| *clear cells | | | | | | | | |

| **Table Q-7a-6:** PBMCs from resistant patients were treated with 1400 μg/ml carboplatin for 0, 3, 6 or 24h, post-incubated in drug-free medium for 0, 1, 2, 6 or 24h and the drug-induced DNA damage was analyzed using comet assay | | | | | | | | |
| --- | --- | --- | --- | --- | --- | --- | --- | --- |
| # | Olive Tail Moment (OTM) | | | | | | | |
|  | 0h treatment  (intrinsic) | 3h treatment | 6h treatment | 24h treatment | | | | |
|  |  |  |  | Post-incubation time | | | | |
|  |  |  |  | 0h | 1h | 2h | 6h | 24h |
| 1* | 3.7 | 6 | 9 | 13 | 12 | 11 | 8.4 | 3 |
| 2 | 7.0 | 11 | 14 | 16 | 15 | 15 | 12.9 | 7 |
| 3 | 16.1 | 14 | 17 | 19 | 18 | 17 | 14.9 | 13 |
| 4 | 4.5 | 10 | 12 | 15 | 14 | 13 | 11.8 | 4 |
| mv | 7.8 | 10.3 | 13.0 | 15.8 | 14.8 | 14.0 | 12.0 | 6.8 |
| max | 16.1 | 14.0 | 17.0 | 19.0 | 18.0 | 17.0 | 14.9 | 13.0 |
| min | 3.7 | 6.0 | 9.0 | 13.0 | 12.0 | 11.0 | 8.4 | 3.0 |
| *clear cells | | | | | | | | |

**Figure Q-7a-1: Kinetics of carboplatin-induced DNA damage formation/repair in PBMCs**. PBMCs from healthy volunteers (HC) and OC patients (SENS, sensitive; RES, resistant) were treated with 1400 μg/ml carboplatin for 0, 3, 6 or 24h, post-incubated in drug-free medium for 0, 1, 2, 6 or 24h and the drug-induced DNA damage was analyzed using immunofluorescence quantification of γH2AX (A) or comet assay (B).

Q-7b. “Thereafter, DNA damage levels were eliminated and the extent of repair was higher in healthy volunteers than in patients. Interestingly, in line with the results from the cell lines experiments, platinum-sensitive patients showed significantly lower repair efficiency than platinum-resistant ones (P<0.03). Particularly, using confocal microscopy the γH2AX foci were removed with t1/2 = 2.7±0.5h in healthy controls, 8.8±1.9h in platinum-resistant and 15.4±3.2h in platinum-sensitive patients. By using comet assay, the t1/2 values for carboplatin-induced DNA damage repair were 4.8±1.4h, 12.9±1.9h and 21.4±2.6h, respectively.”

The data are presented in Tables Q-7a-1, Q-7a-2, Q-7a-3, Q-7a-4, Q-7a-5, Q-7a-6 and Figure Q-7a-1 (above), as well as in Tables Q-5b-1 and Q-5b-2 (Supporting Information, S5 File). Also, see the “*Additional data*” excel file, pages 14 and 15 (Helios Digital Repository).

Q-7c. “in both cell lines DNA damage reached highest levels at the end of the drug treatment, with DNA damage being significantly higher in A2780 than in A2780/C30 cells (data not shown).”

Please, see Tables Q-7c-1, Q-7c-2, Q-7c-3, Q-7c-4 and Figure Q-7c-1 below. See also the “*Additional data - Stefanou et al PLOS ONE 2014*” excel file, pages 8-11 (Helios Digital Repository).

| **Table Q-7c-1:** Sensitive A2780 cells were treated with 100 μg/ml carboplatin for 0, 3, 6 or 24h, post-incubated in drug-free medium for 0, 1, 2, 6 or 24h and the drug-induced DNA damage was analyzed using immunofluorescence quantification of γH2AX | | | | | | | | |
| --- | --- | --- | --- | --- | --- | --- | --- | --- |
| # | % of γH2AX positive cells | | | | | | | |
|  | 0h treatment  (intrinsic) | 3h treatment | 6h treatment | 24h treatment | | | | |
|  |  |  |  | Post-incubation time | | | | |
|  |  |  |  | 0h | 1h | 2h | 6h | 24h |
| 1 | 32 | 48 | 71 | 86 | 83 | 72 | 63 | 23 |
| 2 | 29 | 41 | 62 | 91 | 82 | 80 | 60 | 28 |
| 3 | 27 | 44 | 66 | 94 | 86 | 74 | 59 | 25 |
| 4 | 37 | 53 | 68 | 88 | 82 | 84 | 68 | 31 |
| 5 | 24 | 52 | 65 | 84 | 75 | 81 | 62 | 22 |
| 6 | 24 | 45 | 69 | 72 | 79 | 76 | 67 | 34 |
| mv | 28.8 | 47.2 | 66.8 | 85.8 | 81.2 | 77.8 | 63.2 | 27.2 |
| max | 37.0 | 53.0 | 71.0 | 94.0 | 86.0 | 84.0 | 68.0 | 34.0 |
| min | 24.0 | 41.0 | 62.0 | 72.0 | 75.0 | 72.0 | 59.0 | 22.0 |

| **Table Q-7c-2:** Resistant A2780/C30 cells were treated with 100 μg/ml carboplatin for 0, 3, 6 or 24h, post-incubated in drug-free medium for 0, 1, 2, 6 or 24h and the drug-induced DNA damage was analyzed using immunofluorescence quantification of γH2AX | | | | | | | | |
| --- | --- | --- | --- | --- | --- | --- | --- | --- |
| # | % of γH2AX positive cells | | | | | | | |
|  | 0h treatment  (intrinsic) | 3h treatment | 6h treatment | 24h treatment | | | | |
|  |  |  |  | Post-incubation time | | | | |
|  |  |  |  | 0h | 1h | 2h | 6h | 24h |
| 1 | 17 | 48 | 58 | 86 | 65 | 68 | 36 | 13 |
| 2 | 18 | 39 | 51 | 78 | 70 | 60 | 27 | 14 |
| 3 | 22 | 45 | 49 | 71 | 75 | 57 | 37 | 23 |
| 4 | 12 | 43 | 53 | 90 | 69 | 63 | 35 | 18 |
| 5 | 15 | 44 | 55 | 73 | 76 | 65 | 28 | 15 |
| 6 | 20 | 47 | 47 | 79 | 73 | 66 | 40 | 14 |
| mv | 17.3 | 44.3 | 52.2 | 79.5 | 71.3 | 63.2 | 33.8 | 16.2 |
| max | 22.0 | 48.0 | 58.0 | 90.0 | 76.0 | 68.0 | 40.0 | 23.0 |
| min | 12.0 | 39.0 | 47.0 | 71.0 | 65.0 | 57.0 | 27.0 | 13.0 |

Similar results were obtained using comet assay.

| **Table Q-7c-3:** Sensitive A2780 cells were treated with 100 μg/ml carboplatin for 0, 3, 6 or 24h, post-incubated in drug-free medium for 0, 1, 2, 6 or 24h and the drug-induced DNA damage was analyzed using comet assay | | | | | | | | |
| --- | --- | --- | --- | --- | --- | --- | --- | --- |
| # | Olive Tail Moment (OTM) | | | | | | | |
|  | 0h treatment  (intrinsic) | 3h treatment | 6h treatment | 24h treatment | | | | |
|  |  |  |  | Post-incubation time | | | | |
|  |  |  |  | 0h | 1h | 2h | 6h | 24h |
| 1 | 28.0 | 37 | 55.0 | 49 | 48.0 | 45.0 | 44.0 | 27.0 |
| 2 | 37.0 | 46 | 57.0 | 58 | 53.0 | 47.0 | 41.0 | 34.0 |
| 3 | 35.0 | 39 | 44.0 | 55 | 55.0 | 48.0 | 41.0 | 30.0 |
| 4 | 26.0 | 48 | 48.0 | 61 | 47.0 | 50.0 | 46.0 | 26.0 |
| 5 | 39.0 | 40 | 41.0 | 52 | 49.0 | 49.0 | 43.0 | 30.0 |
| 6 | 38.0 | 37 | 44.0 | 47 | 55.0 | 53.0 | 44.0 | 31.0 |
| mv | 33.8 | 41.2 | 48.2 | 53.7 | 51.2 | 48.7 | 43.2 | 29.7 |
| max | 39.0 | 48.0 | 57.0 | 61.0 | 55.0 | 53.0 | 46.0 | 34.0 |
| min | 26.0 | 37.0 | 41.0 | 47.0 | 47.0 | 45.0 | 41.0 | 26.0 |

| **Table Q-7c-4:** Resistant A2780/C30 cells were treated with 100 μg/ml carboplatin for 0, 3, 6 or 24h, post-incubated in drug-free medium for 0, 1, 2, 6 or 24h and the drug-induced DNA damage was analyzed using comet assay | | | | | | | | |
| --- | --- | --- | --- | --- | --- | --- | --- | --- |
| # | Olive Tail Moment (OTM) | | | | | | | |
|  | 0h treatment  (intrinsic) | 3h treatment | 6h treatment | 24h treatment | | | | |
|  |  |  |  | Post-incubation time | | | | |
|  |  |  |  | 0h | 1h | 2h | 6h | 24h |
| 1 | 15.0 | 20 | 27.0 | 45 | 41.0 | 29.0 | 19.0 | 9.0 |
| 2 | 11.0 | 28 | 33.0 | 39 | 30.0 | 34.0 | 28.0 | 14.0 |
| 3 | 19.0 | 29 | 31.0 | 35 | 35.0 | 39.0 | 29.0 | 12.0 |
| 4 | 15.0 | 24 | 29.0 | 37 | 37.0 | 38.0 | 26.0 | 16.0 |
| 5 | 16.0 | 26 | 35.0 | 38 | 39.0 | 35.0 | 31.0 | 15.0 |
| 6 | 10.0 | 22 | 36.0 | 41 | 38.0 | 33.0 | 27.0 | 11.0 |
| mv | 14.3 | 24.8 | 31.8 | 39.2 | 36.7 | 34.7 | 26.7 | 12.8 |
| max | 19.0 | 29.0 | 36.0 | 45.0 | 41.0 | 39.0 | 31.0 | 16.0 |
| min | 10.0 | 20.0 | 27.0 | 35.0 | 30.0 | 29.0 | 19.0 | 9.0 |

**Figure Q-7c-1**: **Kinetics of carboplatin-induced DNA damage formation/repair in OC cell lines.**  Sensitive A2780 and resistant A2780/C30 cells were treated with 100 μg/ml carboplatin for 0, 3, 6 or 24h, post-incubated in drug-free medium for 0, 1, 2, 6 or 24h and the drug-induced DNA damage was analyzed using immunofluorescence quantification of γH2AX (A) or comet assay (B).

Q-7d. “Optimal results were obtained following 48h incubation of PBMCs with 10μg/ml PHA (data not shown).”

Tables Q-7d-1, Q-7d-2, Q-7d-3, Q-7d-4 and Figure Q-7d-1 below show the data that support our conclusions. Also, see the “*Additional data*” excel file, pages 23-26 (Helios Digital Repository).

| **Table Q-7d-1:** The levels of DNA damage in PBMCs from 9 healthy volunteers treated with 150 μg/ml cisplatin for 3h, then incubated in drug-free medium for 0, 6 or 24h and analyzed using immunofluorescence quantification of γH2AX. No PHA stimulation | | | | |
| --- | --- | --- | --- | --- |
| post-incubation time (h) | % of γH2AX positive cells | | | |
|  | no cisplatin treatment | | cisplatin-treated | |
|  | mv | Sd | mv | sd |
| 0/0* | 5.7 | 1.4 | 6.0 | 1.4 |
| 0 | 4.7 | 1.5 | 6.7 | 1.3 |
| 6 | 4.3 | 1.0 | 7.7 | 1.4 |
| 24 | 5.3 | 1.3 | 8.3 | 1.9 |
| *baseline | | | | |

| **Table Q-7d-2:** The levels of DNA damage in PBMCs from 9 healthy volunteers stimulated into proliferation using 10 μg/ml PHA for 24h, treated with 150 μg/ml cisplatin for 3h, then incubated in drug-free medium for 0, 6 or 24h and analyzed using immunofluorescence quantification of γH2AX | | | | |
| --- | --- | --- | --- | --- |
| post-incubation time (h) | % of γH2AX positive cells | | | |
|  | no cisplatin treatment | | cisplatin-treated | |
|  | mv | Sd | mv | sd |
| 0/0* | 5.3 | 1.7 | 6.3 | 1.8 |
| 0 | 5.7 | 1.5 | 8.3 | 2.3 |
| 6 | 6.7 | 1.6 | 8.7 | 1.2 |
| 24 | 6.3 | 1.0 | 13.3 | 1.7 |
| *baseline | | | | |

| **Table Q-7d-3:** The levels of DNA damage in PBMCs from 9 healthy volunteers stimulated into proliferation using 10 μg/ml PHA for 48h, treated with 150 μg/ml cisplatin for 3h, then incubated in drug-free medium for 0, 6 or 24h and analyzed using immunofluorescence quantification of γH2AX | | | | |
| --- | --- | --- | --- | --- |
| post-incubation time (h) | % of γH2AX positive cells | | | |
|  | no cisplatin treatment | | cisplatin-treated | |
|  | mv | Sd | mv | sd |
| 0/0* | 4.3 | 1.2 | 6.7 | 1.9 |
| 0 | 6.3 | 1.2 | 23.3 | 3.4 |
| 6 | 6.7 | 2.0 | 31.7 | 3.3 |
| 24 | 4.7 | 1.5 | 44.3 | 4.3 |
| *baseline | | | | |

| **Table Q-7d-4:** The levels of DNA damage in PBMCs from 9 healthy volunteers stimulated into proliferation using 10 μg/ml PHA for 72h, treated with 150 μg/ml cisplatin for 3h, then incubated in drug-free medium for 0, 6 or 24h and analyzed using immunofluorescence quantification of γH2AX | | | | |
| --- | --- | --- | --- | --- |
| post-incubation time (h) | % of γH2AX positive cells | | | |
|  | no cisplatin treatment | | cisplatin-treated | |
|  | mv | Sd | mv | sd |
| 0/0* | 9.7 | 2.3 | 10.3 | 2.0 |
| 0 | 12.3 | 2.4 | 17.3 | 1.9 |
| 6 | 14.7 | 2.3 | 25.3 | 3.0 |
| 24 | 18.3 | 2.5 | 16.7 | 2.2 |
| *baseline | | | | |

**Figure Q-7d-1: The effect of PHA stimulation on the DNA damage in PBMCs**. DNA damage in PBMCs from nine healthy volunteers stimulated into proliferation using 10 μg/ml PHA for 0h (A), 24h (B), 48h (C) or 72h (D), treated with 150 μg/ml cisplatin for 3h, then incubated in drug-free medium for 0, 6 or 24h and analyzed using immunofluorescence quantification of γH2AX.

Q-7e. “That is, in all individuals analyzed DNA damage reached highest levels at the end of the drug treatment, with DNA damage being higher in OC patients than in healthy volunteers; platinum-sensitive patients showed higher levels of DNA damage than platinum-resistant ones (data not shown).”

Please see Tables Q-7a-1, Q-7a-2, Q-7a-3, Q-7a-4, Q-7a-5, Q-7a-6 and Figure Q-7a-1 (above).
